# Harmine selectively enhances human beta cell identity markers and function via protein kinase A pathways

**DOI:** 10.1007/s00125-026-06857-5

**Published:** 2026-09-21

**Authors:** Peng Wang, Kunal Kumar, Luca Lambertini, Hongtao Liu, Olivia Wood, Shuxuan Chen, Aidan Pillard, Donald K. Scott, Joao A. Paulo, Steven P. Gygi, Susmita Khamrui, Stefan Mueller, Adolfo Garcia-Ocana, Esra Karakose, Michael B. Lazarus, Robert J. DeVita, Andrew F. Stewart

**Affiliations:** 1 Diabetes Obesity Metabolism Institute, Icahn School of Medicine at Mount Sinai, New York, NY USA; 2 Department of Medicine, Icahn School of Medicine at Mount Sinai, New York, NY USA; 3 Department of Pharmacological Sciences, Icahn School of Medicine at Mount Sinai, New York, NY USA; 4 Drug Discovery Institute, Icahn School of Medicine at Mount Sinai, New York, NY USA; 5 Harvard Medical School, Cambridge, MA USA; 6 Evotec GmbH, Munich, Germany; 7 Arthur Riggs Diabetes and Metabolism Research Institute, City of Hope, Duarte, CA USA

**Keywords:** Beta cell identity, Beta cells, Diabetes, DYRK1A, GLP-1 receptor, Harmine, Human islets, Insulin secretion, PRKAR1A, Protein kinase A

## Abstract

**Aims/hypothesis:**

Harmine and other small-molecule inhibitors of the kinase, dual tyrosine-regulated kinase 1A (DYRK1A), induce human beta cells to replicate and regenerate in vitro and in vivo and are effective at reversing diabetes in animal models. In addition to its beta cell proliferative and regenerative effects, which result from inhibition of DYRK1A (‘Target 1’), harmine also induces expression of essential transcription factors and other genes involved in beta cell identity and function, exemplified by *PDX1*, *MAFA*, *NKX6.1*, *MAFB*, *SIX3*, *SLC2A2*, *ENTPD3* and others. Harmine also rapidly enhances glucose-stimulated insulin secretion in vitro and in vivo. These beta cell identity-enhancing effects are not due to DYRK1A inhibition. We hypothesise that they reflect interactions of harmine with a second target (‘Target 2’). We aimed to identify Target 2.

**Methods:**

We performed an in-depth analysis of multiple different small-molecule inhibitors of DYRK1A in human pancreatic islets using gene expression, single-cell RNA-seq, proliferation, immunocytochemistry of beta cell transcription factors and identity markers, and solid-phase precipitation combined with proteomics.

**Results:**

All small-molecule DYRK1A inhibitors studied (harmine, 2-2c, 5-IT, INDY, leucettine, CC-401, GNF4877) induced human beta cell proliferation via inhibition of DYRK1A. In contrast, silencing DYRK1A had no effect on beta cell transcription factors and identity markers. Further, harmine, 2-2c and 5-IT but not INDY, leucettine, CC-401 or GNF4877 enhanced expression of beta cell phenotypic markers. The enhanced expression of beta cell identity markers resulted from an unexpected activation by harmine of an as-yet unidentified Target 2 that activates the protein kinase A (PKA) pathway.

**Conclusions/interpretation:**

These findings make it clear that all DYRK1A inhibitors are not interchangeable. Those that drive both beta cell proliferation and identity/function will likely be preferable for diabetes therapy. Unravelling the mechanisms controlling the complex harmine–PKA–DYRK1A interactome will provide fruitful ground for future studies.

**Graphical Abstract:**

**Figure:**
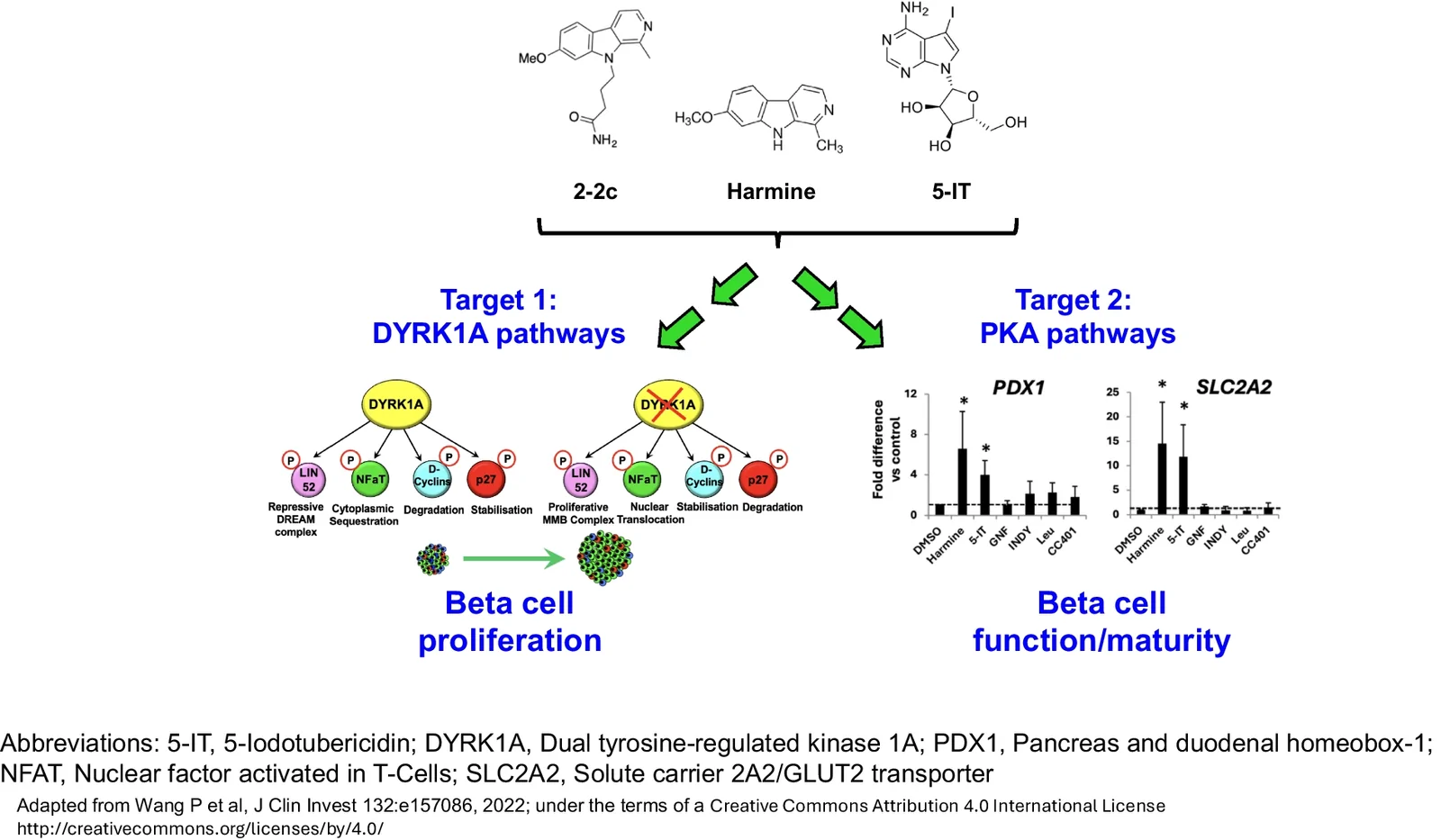

**Supplementary Information:**

The online version of this article (https://doi.org/10.1007/s00125-026-06857-5) contains peer-reviewed but unedited supplementary material.

**Figure:**
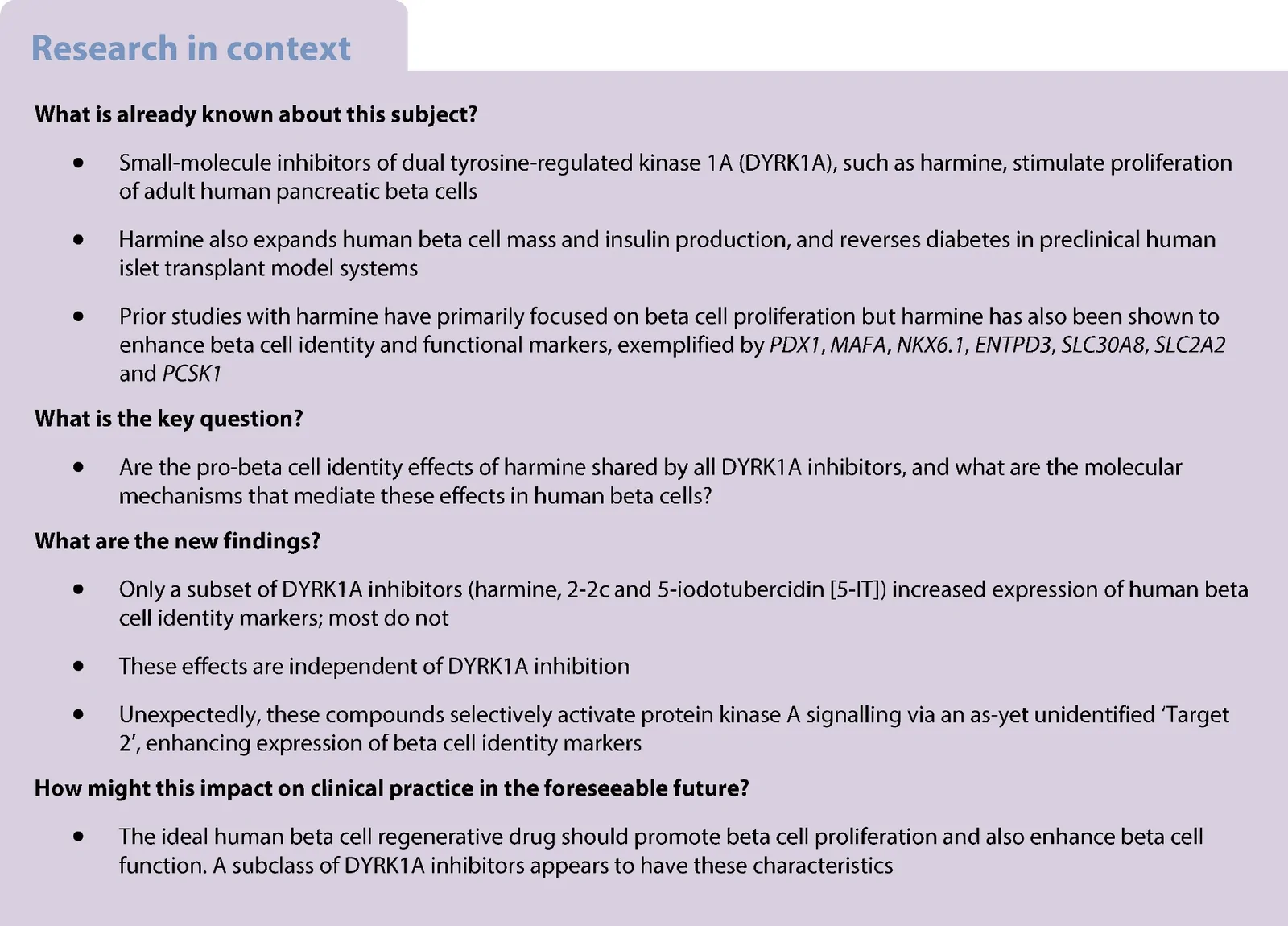

## Introduction

**Fig. 1:**
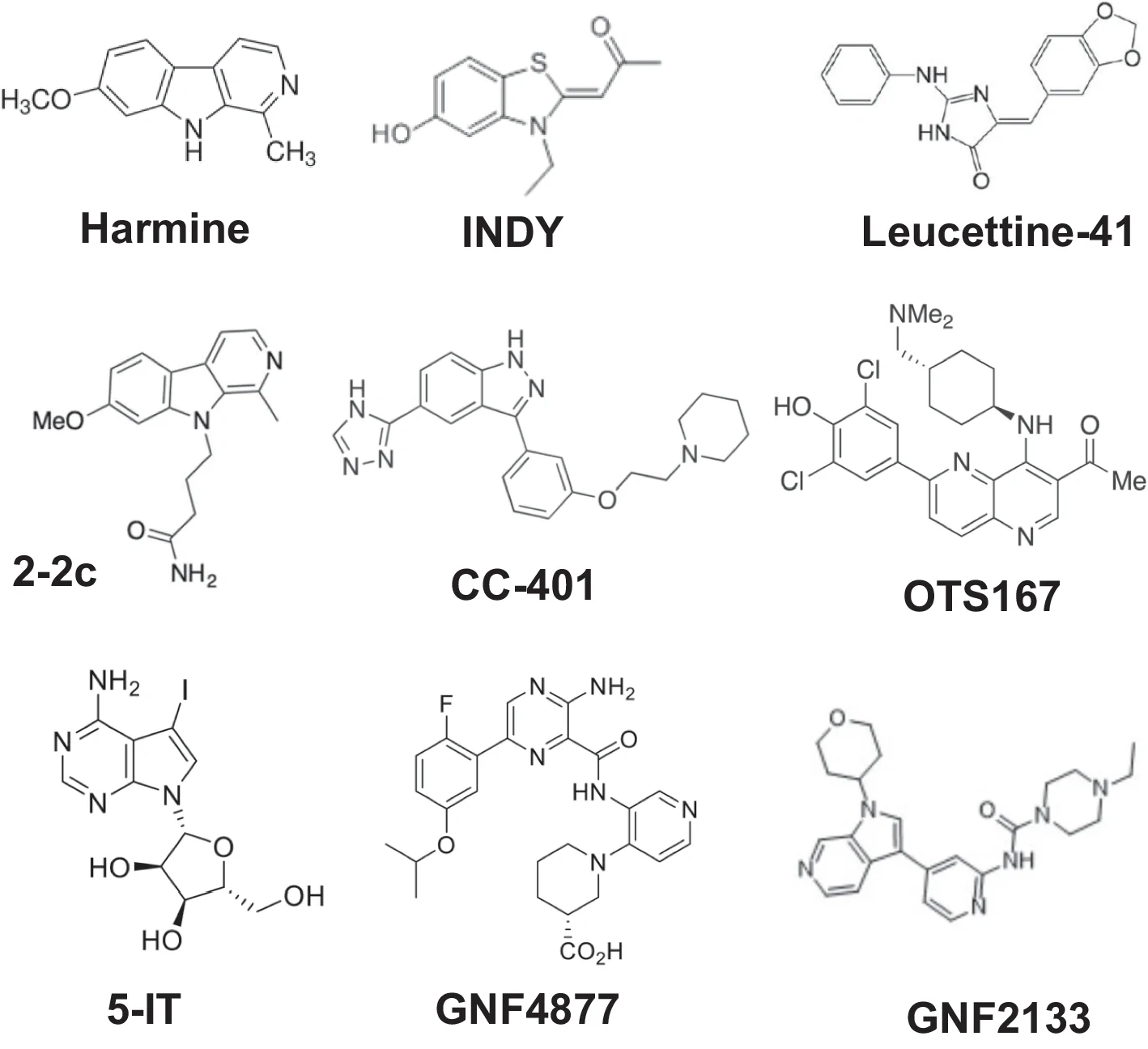
Structures of DYRK1A inhibitors commonly used in beta cell regeneration research. Harmine, 2-2c, 5-IT, GNF4877, INDY, leucettine-41, and CC-401 were evaluated experimentally in this study. GNF2133 and OTS167 are included to illustrate the broader landscape of reported DYRK1A inhibitors but were not investigated further because they were not commercially available during the course of this study

Diabetes affects 500 million people globally [1–4]. While insulin resistance is an underlying driver of type 2 diabetes, both type 1 and type 2 diabetes ultimately result from inadequate production of insulin by the insulin-producing beta cells in the pancreas [5–9]. This beta cell anatomical and functional deficiency has prompted efforts to restore beta cell mass through transplantation of the whole pancreas, isolated pancreatic islets and stem cell-derived human beta cells [9–14]. As an alternative, several laboratories have attempted to develop drugs capable of regenerating the beta cells that remain in most people with diabetes. Our group [15–23] and others [24–31] have focused on developing small-molecule inhibitors of dual tyrosine-regulated kinase (DYRK) 1A (Fig. 1). These small molecules increase human beta cell proliferation in vitro and in vivo [15–31], and markedly expand human beta cell mass in human islet transplant model systems [19]. The human beta cell proliferative effects require inhibition of DYRK1A [15–17, 21, 24], an action which is exerted by all of the molecules shown in Fig. 1.

An additional unanticipated and important beneficial effect of harmine is that it also rapidly (days) enhances expression of human beta cell phenotypic markers, including transcription factors such as PDX1, MafA and NKX6.1, as well as key functional genes including *SLC2A2* (*GLUT2*), *GLP1R* and *PCSK1,* and these events occur at both the mRNA and protein levels [16, 18, 19]. In addition, it is clear that harmine also enhances human beta cell function, including in vitro glucose-stimulated insulin secretion (GSIS) [16]. The enhanced functional effect also translates to the in vivo setting. For example, in experiments using a marginal mass of human islets transplanted into streptozocin-induced diabetic, immunodeficient mice, harmine normalises blood glucose within days [15, 19], long before beta cell numbers increase. This is associated with increases in circulating human insulin concentrations and normalisation of in vivo glucose tolerance testing [15, 19]. Thus, overall, complementing its ability to enhance expression of human beta cell transcription factors and phenotypic markers, harmine also rapidly enhances human beta cell functional responses to glucose in vitro and in vivo [15, 16, 19]. The dual ability to drive both beta cell proliferation and enhance expression of beta cell phenotypic markers makes the DYRK1A inhibitor drug class members attractive potential candidates for treatment of both type 1 diabetes and type 2 diabetes.

Harmine has been widely used as a DYRK1A inhibitor, and its proliferative and function-enhancing effects in beta cells have generally been attributed to DYRK1A inhibition. However, whether these effects are shared across other DYRK1A inhibitors remains unclear. In addition, the extent to which beta cell phenotype and identity are regulated through DYRK1A-dependent vs DYRK1A-independent mechanisms has not been defined. These questions raise the possibility that additional targets or pathways may contribute to the functional effects observed with harmine and related compounds. Here, we explore whether there is only one therapeutically relevant harmine target, DYRK1A (‘Target 1’) or whether there is a second beta cell phenotype-regulating target (‘Target 2’).

## Methods

Detailed experimental procedures are provided in electronic supplementary material (ESM) Methods.

### Human islets

Isolated, de-identified human pancreatic islets from non-diabetic organ donors were obtained through the NIH Integrated Islet Distribution Program (IIDP), Prodo Laboratories, the Alberta Diabetes Institute and the University of Chicago. Donor characteristics and islet preparation details are provided in the ESM Human islet checklist. In total, 91 human islet preparations were studied. Human islets from multiple independent donors were used for each experiment to minimise donor-specific variability. For each donor preparation, aliquots of islets were distributed across vehicle and treatment groups so that all treatment conditions were performed using cells from the same donor, allowing each donor to serve as its own matched biological control. No formal randomisation procedure was used. Investigators were not blinded to treatment allocation or outcome assessment. No predefined exclusion criteria were applied other than technical failure or inadequate sample quality (e.g. poor cell viability or insufficient RNA or protein yield). No donor preparations or experimental results were excluded on the basis of study outcome. When technical failures occurred, experiments were repeated using additional donor preparations.

Human islets from both male and female non-diabetic donors were included where available. Individual experiments involved relatively small numbers of donor preparations and were designed to evaluate treatment effects using donor-matched comparisons rather than biological sex as an experimental variable.

### Chemicals

Accutase (Mediatech, Manassas, VA, USA), harmine (Sigma, St Louis, MO, USA), leucettine-41 (Adipogen, San Diego, CA, USA), INDY and 5-iodotubercidin (5-IT) (Tocris, Bristol, UK), GNF4877 and CC-401 (MedChemExpress, Monmouth Junction, NJ, USA), forskolin (Selleckchem, Houston, TX, USA) and H89 (MedChemExpress) were used. Compound 2-2c was synthesised at the Drug Discovery Institute and the Department of Pharmacological Sciences at The Icahn School of Medicine at Mount Sinai.

### Adenoviruses and transduction

Adenoviral constructs for gene silencing or overexpression were generated using standard methods as previously described [19]. Adenoviruses targeting DYRK family members, DREAM complex components, CDC-like kinases (CLKs), NFAT constructs, *PRKAR1A* shRNA and V5-tagged *PRKACA* were used. Detailed cloning strategies and viral constructs are described in ESM Methods [17, 19, 21].

### Human islet dispersion and virus infection

Human islets were dispersed using Accutase and plated on poly-d-lysine/laminin-coated coverslips. Adenoviral transduction was performed for 2 h followed by addition of fresh medium. Cells were cultured for 24–96 h as described in the figure legends.

### Compound treatments

Dispersed islet cells were treated with harmine, 2-2c, 5-IT, GNF4877, INDY, leucettine-41, CC-401, forskolin or H89 at concentrations indicated in the figure legends.

Quantitative reverse transcription PCR Gene expression was assessed by quantitative reverse-transcription PCR (RT-PCR). Primer sequences are listed in ESM Table 1.

### Immunoblotting and Immunocytochemistry

Protein expression was analysed by immunoblotting and immunocytochemistry. Proteins analysed included C-peptide, phospho-CREB, total CREB, PDX1, MafA, NKX6.1, V5, PRKAR1A and GAPDH.

### Human islet proteomics and pulldown assays

Human islet proteomics and pulldown experiments using immobilised harmine analogues were performed.

### Protein purification for thermal shift assay

Recombinant PRKAR1A and PRKACA proteins were purified and analysed by SYPRO-Orange thermal shift assays to assess interactions with harmine, cAMP or ATP.

### Statistics

Statistical analyses were performed using GraphPad Prism version 10.2.0 (GraphPad Software, San Diego, CA, USA). Because human islets from different donors exhibit inter-donor variability, analyses used donor-matched approaches, with each donor serving as an independent biological replicate.

For qPCR experiments, raw C_t_ values were normalised to a reference gene to obtain ΔC_t_ values, and ΔΔC_t_ values were calculated relative to control (DMSO). Statistical analyses were performed on ΔC_t_ values, while fold-change values (${2}^{{-\Delta \Delta \mathrm{C}}_{\mathrm{t}}}$) were used for presentation.

Data normality was assessed using the Shapiro–Wilk test. Comparisons between two matched groups used paired two-tailed Student’s *t* tests or Wilcoxon signed-rank tests, as appropriate. Comparisons involving more than two matched groups used repeated-measures one-way ANOVA followed by Dunnett’s multiple comparisons test, or the Friedman test with Dunn’s correction when normality assumptions were not met.

Data are presented as mean ± SEM. *p*<0.05 was considered statistically significant.

## Results

### Harmine, 2-2c and 5-IT uniquely and selectively upregulate human beta cell identity marker genes

**Fig. 2:**
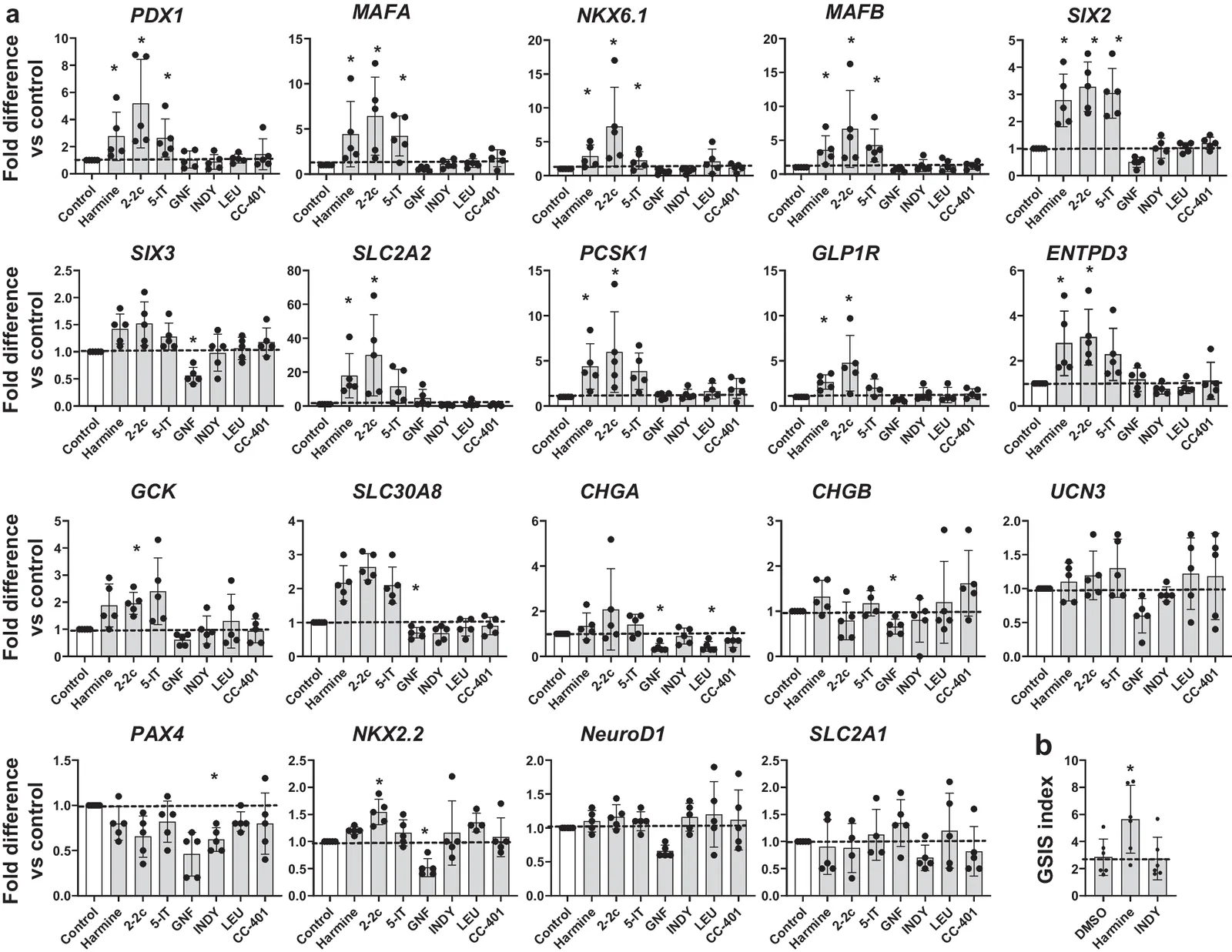
Effects of DYRK1A inhibitors on markers of human beta cell identity and function assessed by quantitative PCR on whole human islets. (**a**) Harmine, 2-2c and 5-IT reproducibly increase most of these markers in human islets, whereas GNF4877, INDY, leucettine-41 and CC-401 fail to do so compared with DMSO control. Not all identity markers are increased by DYRK1A inhibitors, exemplified by *CHGB*, *UCN3*, *PAX4*, *NKX2.2*, *NeuroD1* and *SLC2A1* (*GLUT1*). Doses of each drug were selected as the most effective for human beta cell proliferation in a prior study [17]: harmine 10 µmol/l; 2-2c 3 µmol/l; 5-IT 1 µmol/l; GNF4877 2 µmol/l; INDY 15 µmol/l; leucettine-41 20 µmol/l; and CC-401 10 µmol/l. (**b**) Human islets were treated with DMSO, harmine or INDY for 48 h, followed by GSIS analysis. Insulin secretion was measured at low (2.8 mmol/l) and high (16.8 mmol/l) glucose and normalised to DNA content. GSIS index was calculated as the ratio of insulin secretion at 16.8 mmol/l vs 2.8 mmol/l glucose. Harmine, but not INDY, enhanced GSIS in human islets. Data are shown as mean ± SEM from independent human islet donors. *p<0.05 vs DMSO control (determined by one-way ANOVA followed by Dunnett’s multiple comparisons test). GNF, GNF4877; LEU, leucettine-41

We have previously reported that harmine and 2-2c are able to augment PDX1, NKX6.1 and MafA expression in human islets [16, 19], and this results in augmented human beta cell function and GSIS (Fig. 2b) [16, 19]. Since we had assumed that this was a class effect for all DYRK1A inhibitors, we were surprised to observe that these effects were limited to treatment with harmine, 2-2c and 5-IT (Fig. 2a). In contrast, GNF4877, INDY, leucettine-41 and CC-401, while being tenfold more effective drivers of proliferation [17], all failed to induce human beta cell identity markers (Fig. 2a). Dose–response studies further demonstrated that the proliferation-selective DYRK1A inhibitors INDY and leucettine-41 did not significantly increase beta cell identity gene expression (ESM Fig. 1). Interestingly, beta cell identity marker induction was also selective: while the effect was observed for *PDX1*, *MAFA*, *NKX6.1*, *SLC30A8*, *SLC2A2*, *GLP1R*, *SIX2*, *SIX3*, *PCSK1*, *ENTPD3* and *GCK*, it was not observed for other important markers of beta cell identity and function, such as the chromogranins, *SLC2A1* (encoding GLUT1) and *UCN3* (encoding urocortin-3). ESM Fig. 2 shows that the time course for induction of *PDX1*, *MAFA*, *NKX6.1*, *C2CD4A* and *C2CD4B* was rapid (2–4 h), whereas *SLC2A2, ENTPD3* and *INS* only rose at later time points, consistent with their being downstream responders to *PDX1, MAFA, NKX6.1, C2CD4A* and *C2CD4B*. In contrast, *NEUROD1* and *FOXO1* showed only small and variable responses.

**Fig. 3:**
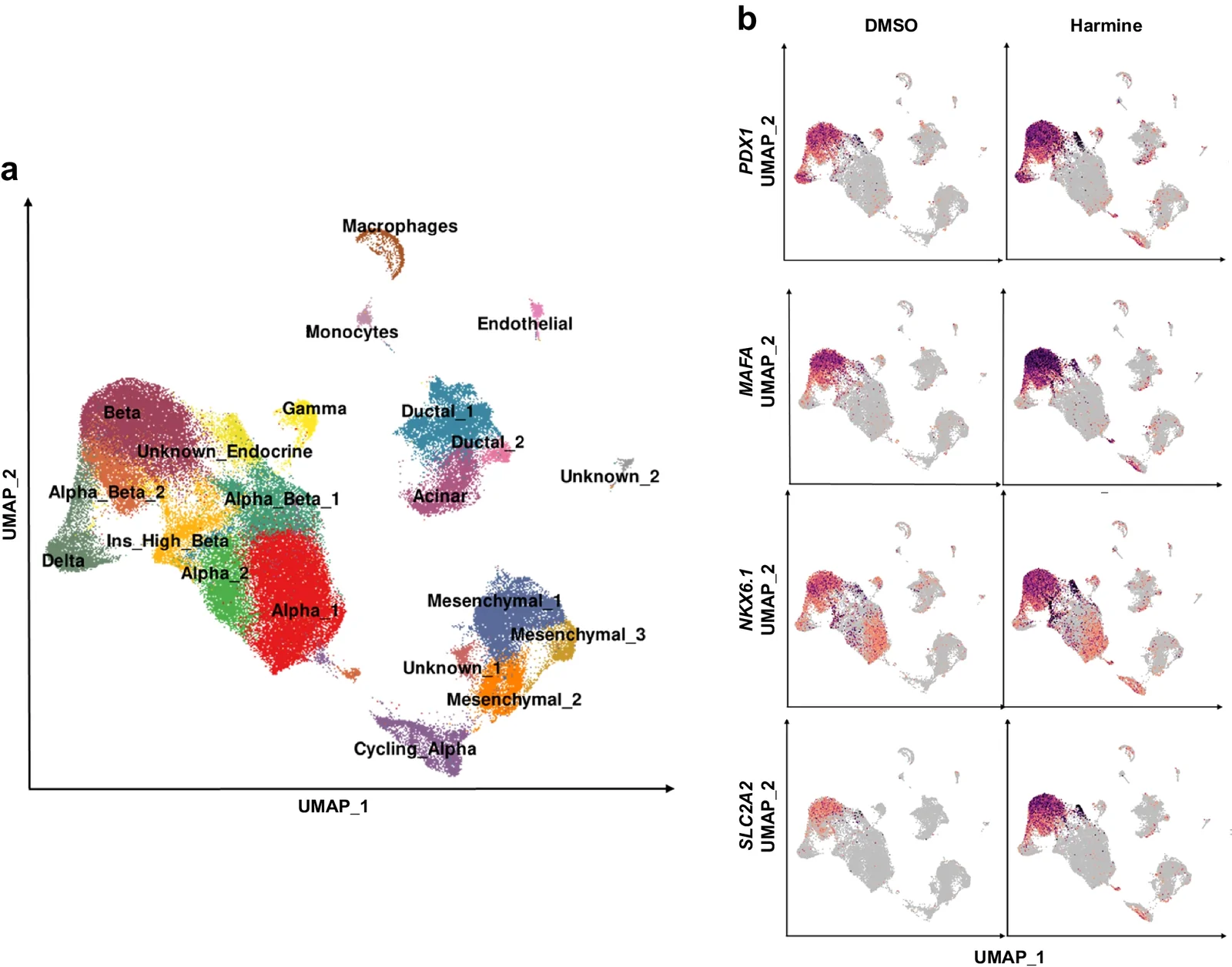
Selected feature plots of single-cell RNA-seq analysis of human islets treated with vehicle or harmine reveal that pro-beta cell identity events occur predominantly, but not exclusively, in beta cells. These feature plots are derived from publicly available datasets from an earlier study of human islets treated for 96 h with vehicle (0.1% DMSO) or 10 µmol/l harmine, then subjected to single-cell RNAseq, all as detailed [18]. *n*=4 human islet preparations for each treatment. (**a**) UMAP showing cell type annotation of all islet cell types. (**b**) Expression of *PDX1*, *MAFA*, *NKX6.1* and *SLC2A2* in response to vehicle vs harmine. Note that in most cases, the major increase in gene expression occurred predominantly in beta cells, whereas in other cases (e.g. *NKX6.1*) the increase was apparent in multiple islet cell types. Colours indicate normalised gene expression levels, with grey representing low or absent expression and purple indicating high expression

The studies depicted in Fig. 2a are whole-islet PCR experiments and thus include all human islet cell types. To determine whether these changes were specific to beta cells or occurred in all islet cell types, we explored our human islet single-cell RNA-seq datasets [18]. As can be seen in Fig. 3, the increases in *PDX1, MAFA* and *SLC2A2* were almost exclusively limited to beta cells, although there was also an increase in delta cells and cells annotated as ‘unknown endocrine cells’. On the other hand, changes in *NKX6.1* involved several other islet endocrine cell types. This wider induction in additional islet cell subtypes also applied to other islet endocrine markers, such as *MAFA*, *MAFB*, *NEUROD1*, *C2CD4A*, *C2CD4B*, *SLC30A8*, *NKX2.2* and *NEUROD1* (not shown).

### Parallel increases in human beta cell proteins

**Fig. 4:**
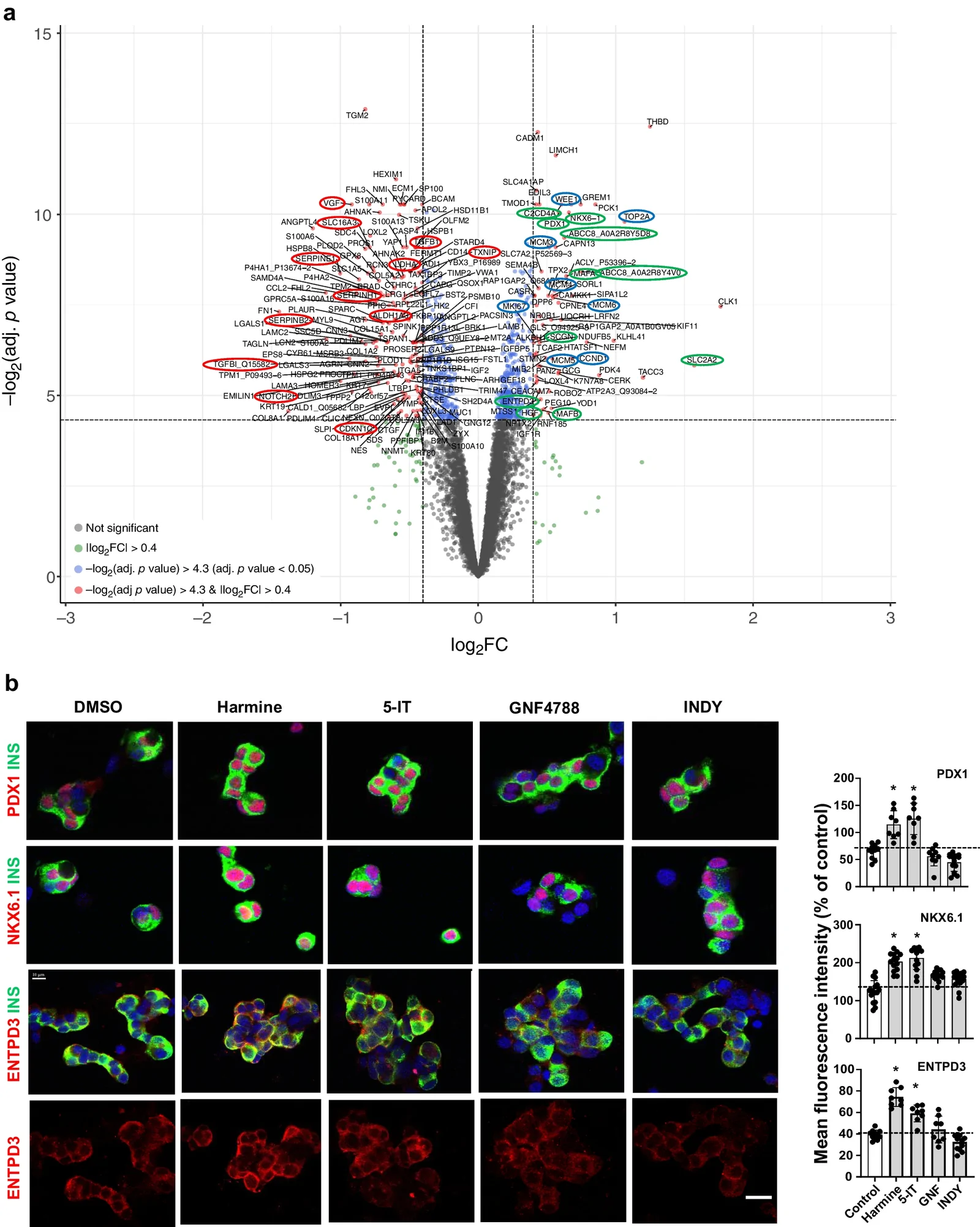
Unbiased proteomics of whole islets from humans and immunocytochemistry in human islet beta cells reveal that changes at the mRNA level translate to the protein level. (**a**) Effects of harmine treatment (10 µmol/l) for 72 h on three preparations of whole islets. Blue circles indicate markers of cell cycle activity increased by harmine treatment, including Ki67, WEE1, TOP2A and CCND1; green circles indicate beta cell identity and functional markers increased by harmine treatment, including PDX1, NKX6.1, MAFA, C2CD4A, SLC2A2, MAFB, ENTPD3, SCGN, ABCC8 and HGF; red circles indicate proteins decreased following harmine treatment, including ALDH1A3, CDKN1C and members of the serpin family. Log_2_ fold change (FC) and adjusted (adj.) *p* values are shown on the *x*- and *y*-axes. For details of the proteins shown in the figure, please see ESM Table 2. (**b**) Immunohistochemistry for insulin, PDX1, NKX6.1 and ENTPD3 and quantification in dispersed human islets treated with the drugs indicated. Note that all three markers were increased by harmine and 5-IT, but not by GNF4877 or INDY. Scale bar, 20 μm. Quantification data are from three individual experiments quantified by Image-J. Data are presented as mean ± SEM with individual donor values shown. Fluorescence intensity was quantified and normalised to control for each donor. *p<0.05 (determined using repeated-measures one-way ANOVA followed by Dunnett’s multiple comparison test comparing each treatment vs control)

To determine whether changes at the mRNA level translated to the proteome, we performed proteomics on human islets treated with harmine or vehicle (0.1% vol./vol. DMSO). As shown visually in the volcano plot in Fig. 4a, and in complete form in ESM Table 2, many of the factors identified at the mRNA level translated to the proteome. For example, PDX1, NKX6.1, MafA, C2CD4A, SLC2A2, MafB, ENTPD3 (marked with green circles in Fig. 4a) were all increased by harmine. Others of interest that we had not specifically queried in Fig. 2 were also increased (SCGN, ABCC8, HGF). In addition, markers of cell cycle activity such as Ki67, WEE1, TOP2A and CCND1 (blue circles in Fig. 4a) were also increased, perhaps as expected. Conversely, harmine treatment was associated with declines in ALDH1A3 (a marker of beta cell stress and dysfunction [32]), CDKN1C (p57^KIP2^, a cell cycle inhibitor important in beta cell cycle arrest, reported to be reduced with harmine treatment in prior studies [18, 33]), and the serpin family, which has been associated with beta cell proliferation [34] (red circles in Fig. 4a). Other examples are shown in Fig. 4a and ESM Table 2. KEGG and GO pathway analyses did not reveal additional novel pathway information. Independent confirmation of the proteomics results at the immunocytochemistry level showed clear and statistically significant increases within beta cell nuclei of PDX1 and NKX6.1 (Fig. 4b). Cell surface abundance of ENTPD3 also was increased on beta cells (Fig. 4b). These findings confirm that events observed at the mRNA level translate to the protein level, and occur in human beta cells.

### The increase in beta cell identity markers is independent of DYRK1A

**Fig. 5:**
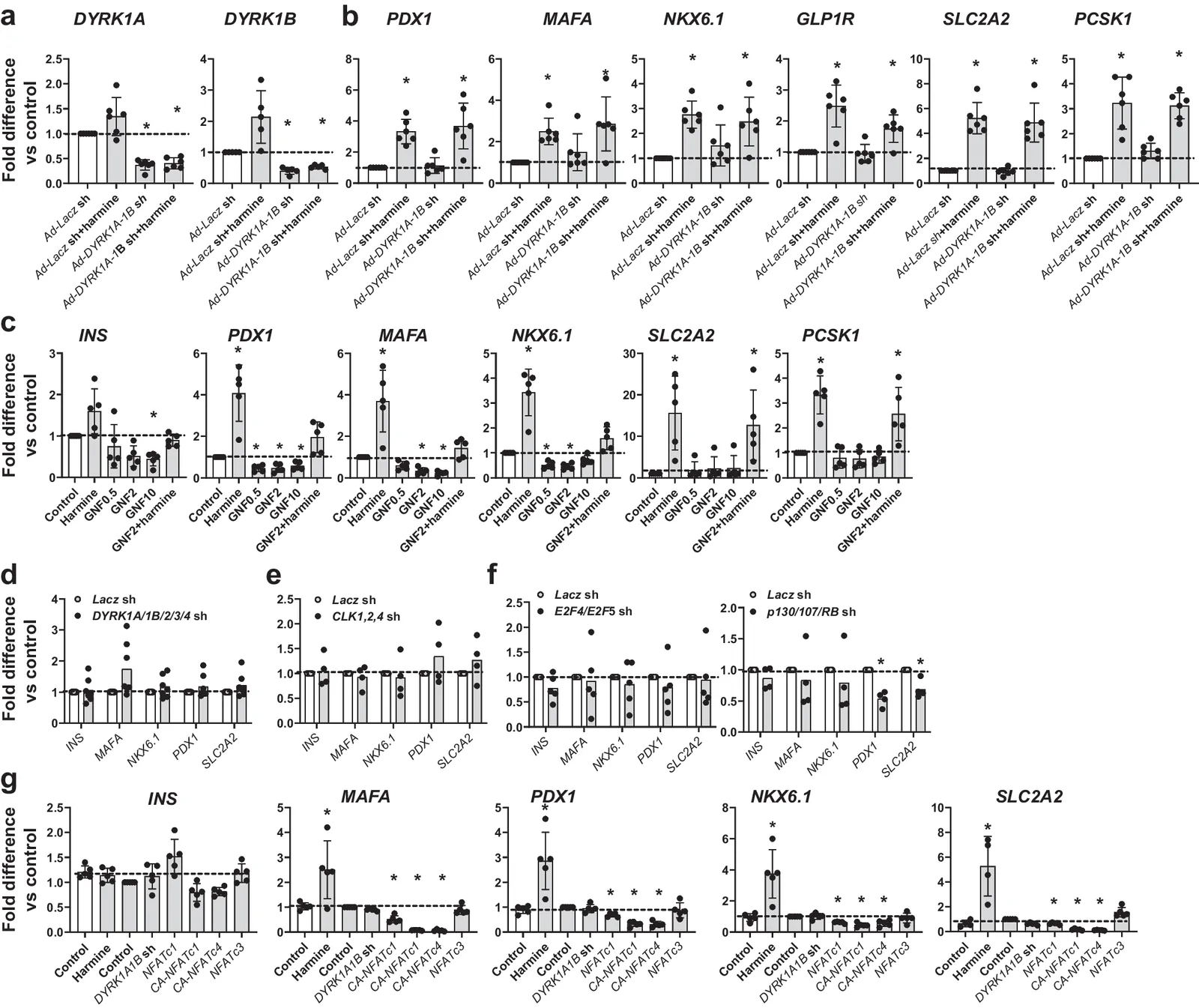
Exploring and eliminating candidates for the pro-beta cell identity effects of harmine. (**a**) Silencing both *DYRK1A* and *DYRK1B* with a single adenovirus is effective in reducing *DYRK1A* and *DYRK1B* mRNA expression by 60–70%, as documented previously [15–17, 21]. (**b**) Silencing *DYRK1A* and *DYRK1B* had no effect on basal identity marker expression. Most importantly, there was no negative effect on harmine-induced increases in these key identity markers. (**c**) The effects of GNF4877 on human islet identity markers. Note that while harmine displayed its usual induction of identity markers, GNF4877 at three different doses (0.5, 2.0 and 10 µmol/l) had no beneficial effects on identity markers and in several instances actually blocked the identity-enhancing effect of harmine. (**d**, **e**) Silencing the entire DYRK family (*DYRK1A*, *DYRK1B*, *DYRK2*, *DYRK3* and *DYRK4*) (**d**) or CLKs 1, 2 and 4 (CLK3 is not expressed in human islets) (**e**) had no effect on human beta cell identity marker expression. (**f**) Silencing of DREAM complex components (E2F4/5) or pocket protein family members (p130/RBL2, p107/RBL1 and RB1) did not affect basal or harmine-induced expression of beta cell identity markers. (**g**) Overexpressing multiple wild-type and constitutively active members of the NFAT transcription factor family, although effective in some systems [36], had no effect on human beta cell identity marker expression. All gene-silencing efficiencies shown in this figure were previously validated and reported [17]. Collectively, these studies make it clear that DYRK1A, DYRK1B, DYRK2, DYRK3, DYRK4, CLKs 1, 2 and 4, and the NFAT family do not mediate, and are not necessary for, the identity-enhancing effects of harmine, and that GNF4877 is a non-specific inhibitor of human beta cell identity marker expression. Fold-change values relative to control are shown for presentation. Statistical analyses were performed on donor-matched ΔC_t_ values. *p<0.05 (determined using repeated-measures one-way ANOVA followed by Dunnett’s multiple comparison test comparing each treatment vs control or paired two-tailed Student’s *t* test for two-group comparisons) for the following comparisons: vs corresponding Ad-LacZ sh control group in the absence or presence of harmine (**a**, **b**); vs untreated control group (**c**); vs the corresponding LacZ sh control group (**d**–**f**); or vs the control adenovirus-treated group (**g**)

Silencing *DYRK1A* mimics the effects of harmine by driving human beta cell proliferation [15, 17, 21, 24]. To ask whether DYRK1A inhibition also was responsible for driving identity marker expression, we simultaneously silenced *DYRK1A* and its homologue *DYRK1B* in human islets and treated the islets with control vehicle (0.1% DMSO) or with harmine. Figure 5a demonstrates that silencing of *DYRK1A* and *DYRK1B* was effective in reducing the expression of the proteins by 75%, a level more than sufficient to drive human beta cell proliferation [15, 17, 21, 24]. In contrast, Fig. 5b shows that combined silencing of *DYRK1A* and *DYRK1B* had no effect on identity marker expression. Critically, harmine was fully able to drive identity marker expression despite *DYRK1A* and *DYRK1B* silencing (Fig. 5b). These findings make it clear that the beta cell identity-enhancing effect of harmine is not likely mediated by, and does not appear to involve, DYRK1A or DYRK1B. Instead, they suggest that harmine has an additional second target that mediates its identity-enhancing effects.

### A candidate approach to identifying the identity-enhancing second target

GNF4877 was one of the first DYRK1A inhibitors shown to drive human beta cell proliferation [27]. Although we and others have confirmed that GNF4877 drives human beta cell proliferation [17], we found that GNF4877 does not increase, but actually inhibits, both basal harmine-induced induction of human beta cell phenotypic markers (Fig. 5c). This is likely explained by its broad non-specificity in targeting hundreds of other kinases [17, 23].

Kinome-wide scans indicate that harmine, 2-2c, 5-IT, GNF4877 and INDY inhibit kinases in addition to DYRK1A, including other DYRK family members and CLKs [17, 23]. Silencing the DYRK and CLK families in human islets produced little or no effect on identity markers (Fig. 5d, e). Because DYRK1A phosphorylation of LIN52 promotes assembly of proliferation repressive DREAM complex, and human beta cell quiescence [21], we explored key members of the DREAM complex (E2Fs 4 and 5, p130 and p107). Although silencing of these genes is effective (ESM Fig. 3) and stimulates beta cell proliferation [21], it had no effect on beta cell phenotypic marker expression (Fig. 5f). We next explored the NFAT transcription factor family, since harmine promotes NFAT nuclear translocation and cell cycle activation [35, 36]. Overexpression of native and constitutively active forms of NFATs (C1, C3, C4) (ESM Fig. 3) had no effect on beta cell phenotypic markers (Fig. 5g). Thus, collectively, our candidate approach failed to identify a mediator of harmine-induced beta cell identity marker expression.

### An unbiased proteomics approach identifies the protein kinase A family as a potential second identity-enhancing target

**Fig. 6:**
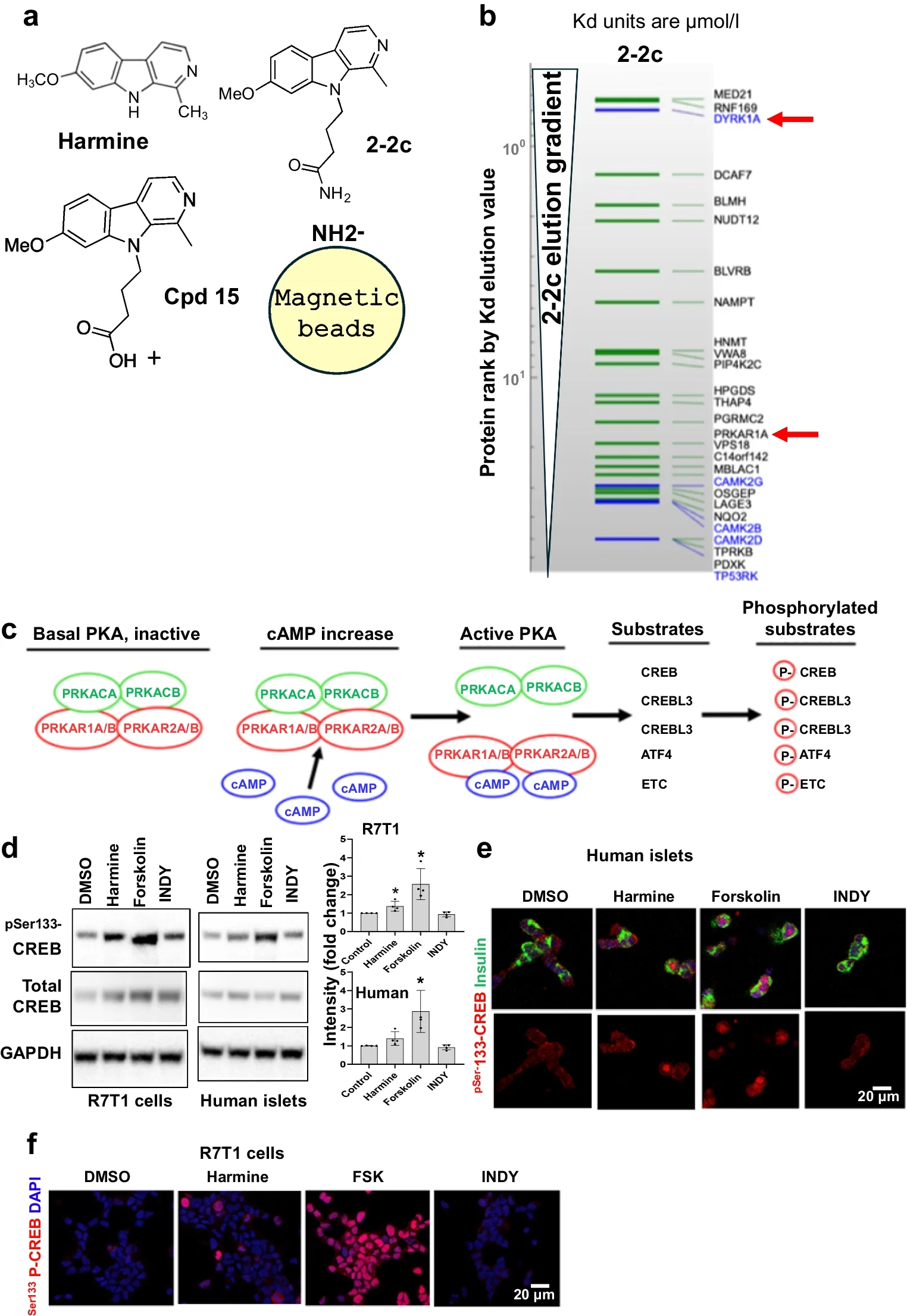
Pulldown of harmine-interacting proteins in human islet extracts reveals protein kinase A interactions. (**a**) Structures of harmine, 2-2c and the new compound 15 (Cpd 15). Note that the tail of 2-2c contains a C-terminal amide group. Converting this amide to a carboxylic acid in new compound Cpd 15, permitted binding of the 2-2c/harmine-like beta carboline to solid-phase magnetic beads. Harmine is the parent β-carboline compound. Cpd 15 is a carboxylated derivative immobilised on magnetic beads for affinity purification, whereas 2-2c is a soluble amide derivative used as the competitive elution compound to release bound proteins according to their binding affinity. (**b**) Human islet extracts were mixed with the solid-phase Cpd 15 and then eluted with a progressive gradient of compound 2-2c, to yield a ranked list of harmine/2-2c-binding proteins in human islets, with the strongest binders displayed at the top. The complete list, in order of affinity, highest to lowest, includes MED21, RNF169, DYRK1A, DCAF7, BLMH, NUDT12, BLVRB, NAMPT, HNMT, VWA8, PIP4K2C, HPGDS, THAP4, PGRMC2, PRKAR1A, VPS18, C14ORF142, MBLAC1, CAMK2G, OSGEP, LAGE3, NQO2, CAMK2B, CAMK2D, TPRKB, PDXK, TP53RK. Blue bars indicate kinases identified in the pulldown assay; green bars indicate non-kinase proteins. Red arrows highlight DYRK1A and PRKAR1A, which were selected for further investigation in this study. (**c**) A cartoon of the protein kinase A heterotetramer, with its association–disassociation and downstream CREB superfamily targets. (**d**) Immunoblots, showing that harmine induces ^133^Ser-CREB phosphorylation in the R7T1 mouse beta cell line and in human islets, as does the cAMP generator forskolin (10 µmol/l), whereas INDY fails to phosphorylate CREB. Bar graphs show quantification from four different experiments. Fold-change values relative to control are shown for presentation. Statistical analyses were performed on donor-matched raw intensity values normalised by internal control. Data are presented as mean ± SEM with individual donor values shown. *p<0.05 for each treatment vs control (determined using repeated-measures one-way ANOVA followed by Dunnett’s multiple comparison test). (**e**) Immunocytochemistry showing that these events occur in human beta cells. Scale bar, 20 μm. (**f**) ^133^Ser-CREB phosphorylation in R7T1 cells at 15 min. A complete time course of harmine and forskolin induction of ^133^SerP-CREB in mouse RT1 cells is shown in ESM Fig. 4

We turned next to an unbiased proteomics approach. We used a modified version of compound 2-2c [21] as bait, and extracts of human islets as a proteome source. Briefly, we generated a tethered analogue of compound 2-2c (Cpd 15, Fig. 6a) and immobilised it on magnetic beads to capture harmine-interacting proteins from human islet lysates. The eluted fractions were then subjected to shotgun proteomics, with results displayed in Fig. 6b.

Reassuringly, DYRK1A was one of the highest affinity binders, confirming the efficacy and specificity of the approach. Other high-affinity eluting proteins were a fragment mediator 21 (MED21), RNF169 and DCAF7. The DYRK1A, DCAF7 and RNF169 signals were reproduced with multiple peptide fragments and in all triplicates. In contrast, the MED21 signal was likely non-specific (only one peptide fragment in only one or three replicates). We next prepared adenoviruses to overexpress and silence RNF169, DCAF7, BLMH, NUDT12 and the remaining eluting proteins shown in Fig. 6b. None had an effect on beta cell identity markers, with the sole exception of PRKAR1A.

Figure 6c shows a model of the protein kinase A (PKA) holoenzyme complex. It is a heterotetramer composed of two catalytic subunits (PRKACA and PRKACB) and two regulatory subunits (PRKAR1A and PRKAR1B) [37]. In human islets, the most abundant members are PRKACA, PRKACB and PRKAR1A. These observations suggest that harmine may act in part by activating the holoenzyme. To explore this possibility, we asked whether harmine could lead to ^133^Ser phosphorylation of the canonical PKA target protein, CREB1. Using both the mouse beta cell line, R7T1 and human islets, we found that harmine effectively leads to ^133^Ser-CREB1 phosphorylation, as does the positive control PKA activator, forskolin (Fig. 6d). Notably, the control DYRK1A inhibitor, INDY, which drives human beta cell proliferation [15, 17, 30] but not beta cell identity genes (Fig. 2), failed to activate PKA (Fig. 6d). These results were confirmed by immunohistochemical labelling of phospho-^133^Ser-CREB1 in both human islets and R7T1 cells (Fig. 6e). ESM Fig. 4 illustrates that the timeframe of PKA activation and CREB phosphorylation in R7T1 cells is rapid (minutes) and persists for hours. Collectively, these studies demonstrate that harmine is able to activate PKA in human and mouse beta cells, establishing PKA engagement as a shared mechanism of this distinct subgroup of DYRK1A inhibitors.

### Manoeuvres that activate PKA mimic harmine-induced pro-beta cell identity effects

**Fig. 7:**
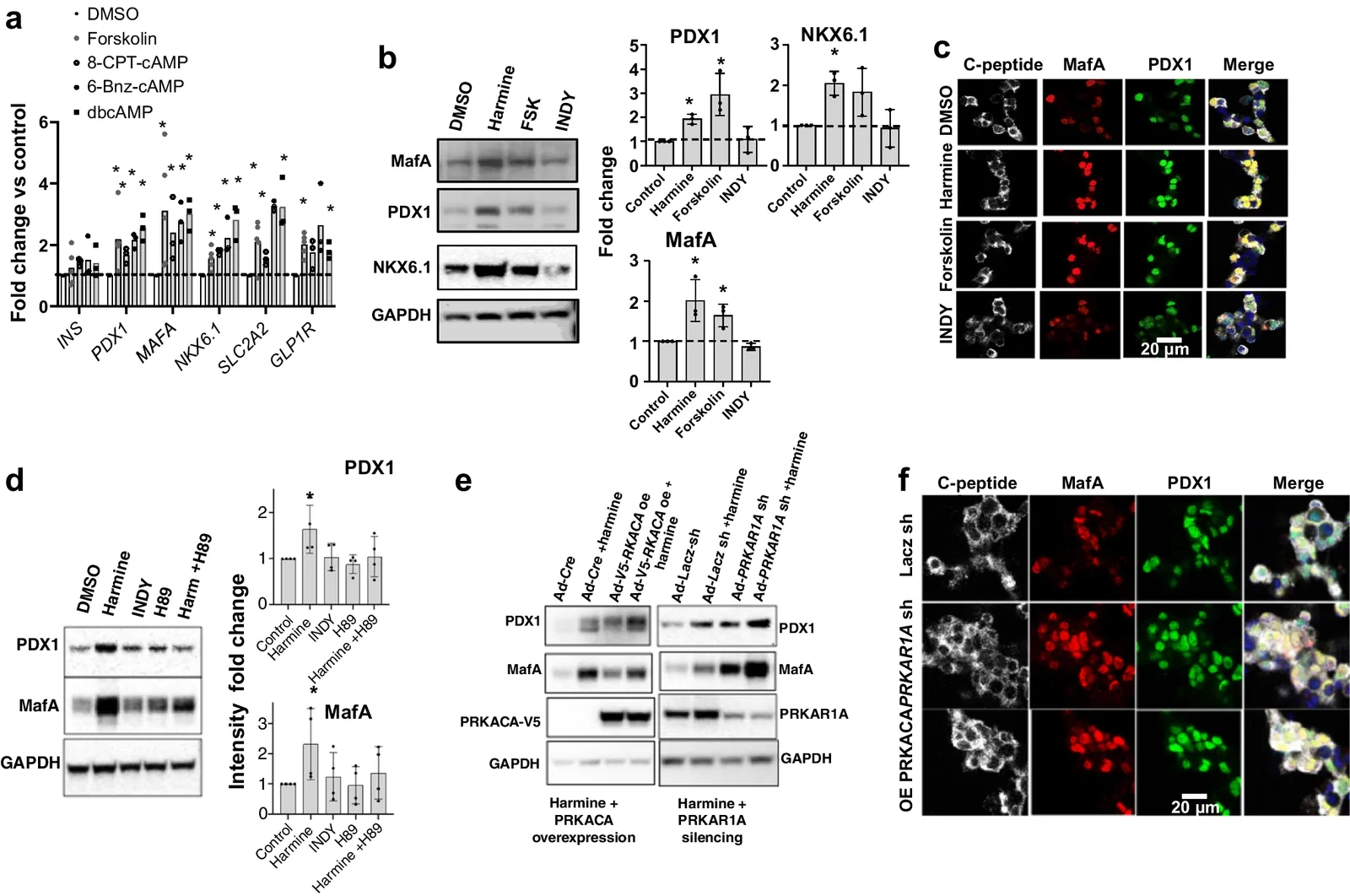
cAMP and PKA signalling activate beta cell identity marker expression in human islets. (**a**) PKA activators, including forskolin (10 mmol/l), 8-CPT-cAMP, 6-Bnz-cAMP and dbcAMP, increased the expression of *MAFA*, *PDX1*, *NKX6.1* and *SLC2A2* mRNA, mimicking the effects of harmine. Fold-change values relative to control are shown for presentation. Statistical analyses were performed on donor-matched ΔC_t_ values. *p<0.05 (determined using repeated-measures one-way ANOVA followed by Dunnett’s multiple comparison test comparing each treatment vs control). (**b**) Immunoblots of human islet treated with vehicle (DMSO), harmine, forskolin or INDY. Harmine and forskolin, but not INDY, increased MafA, PDX1 and NKX6.1 abundance. Bar graphs show fold-change relative to control from four experiments. (**c**) Immunocytochemistry of human islets treated with vehicle, harmine, forskolin or INDY, labelled for C-peptide, MafA and PDX1. Scale bar, 20 μm. (**d**) Immunoblots of human islets treated with vehicle, harmine, INDY, the PKA inhibitor H89 (15 µmol/l), or harmine + H89. Harmine, but not INDY, induced PDX1 and MafA and this effect was blocked by PKA inhibition. Bar graphs show fold-change values relative to control from four experiments. (**e**) Immunoblots showing that overexpression of the catalytic subunit of PKA, PRKACA, with a V5 epitope tag, increased the abundance of PDX1 and MafA in human islets, and this effect was further enhanced by the addition of harmine. Furthermore, silencing the PKA inhibitory regulatory subunit gene *PRKAR1A* mimics, and is enhanced by, the effects of harmine on PDX1 and MafA in human islets. (**f**) Immunocytochemistry showing that silencing *PRKAR1A* or overexpressing PRKACA in human islets increased expression of PDX1 and MafA within beta cells. Scale bar, 20 μm. Statistical analyses were performed on donor-matched raw intensity values. Data are presented as mean ± SEM with individual donor values shown. *p<0.05 (determined using repeated-measures one-way ANOVA followed by Dunnett’s multiple comparison test comparing each treatment vs control). FSK, forskolin; oe, overexpression; sh, short hairpin

If harmine acts via PKA to enhance beta cell identity, then other PKA activators should mimic the effects of harmine. To test this hypothesis, we asked whether the adenylyl cyclase activator forskolin could augment beta cell identity marker expression. Figure 7a shows that this is the case, with forskolin increasing expression of *MAFA, PDX1, NKX6.1* and *SLC2A2* mRNA in human islets, exactly like harmine (see Fig. 2). Both immunoblot and immunocytochemistry (Fig. 7b, c) showed that this also occurs at the protein level in human beta cells. In addition to forskolin, multiple pharmacological activators of the cAMP–PKA pathway similarly increased expression of beta cell identity markers, indicating that activation of the PKA axis is sufficient to phenocopy the beta cell identity component of harmine signalling (Fig. 7a). Importantly, INDY, did not share this pro-beta cell identity effect (Fig. 7b, c). Equally importantly, the beta cell identity effects of harmine in human islets were blocked by the PKA inhibitor H89 (Fig. 7d), providing additional documentation, along with CREB phosphorylation, that the beta cell identity-enhancing effect of harmine is mediated by PKA.

### Manipulating individual PKA subunits mimics the effects of harmine

To explore the role of the PKA subunits shown in Fig. 6c more deeply, we prepared adenoviruses to overexpress the gene encoding the constitutively active catalytic subunit PRKACA, or to silence the gene encoding the PKA inhibitory regulatory subunit PRKAR1A, which we had pulled down with harmine (Fig. 6b). Remarkably, overexpression of the active catalytic subunit PRKACA led to increases in PDX1 and MafA (Fig. 7e), comparable with the effect induced by harmine, and this was further enhanced by harmine. More importantly, silencing the inhibitory, regulatory PKA subunit PRKAR1A also induced PDX1 and MafA expression in human islets, similarly to harmine, and this was further enhanced by the addition of harmine (Fig. 7e). Finally, these studies in whole human islets were independently confirmed to occur in human beta cells by immunocytochemistry (Fig. 7f).

### The interaction between harmine and PRKAR1A is indirect

**Fig. 8:**
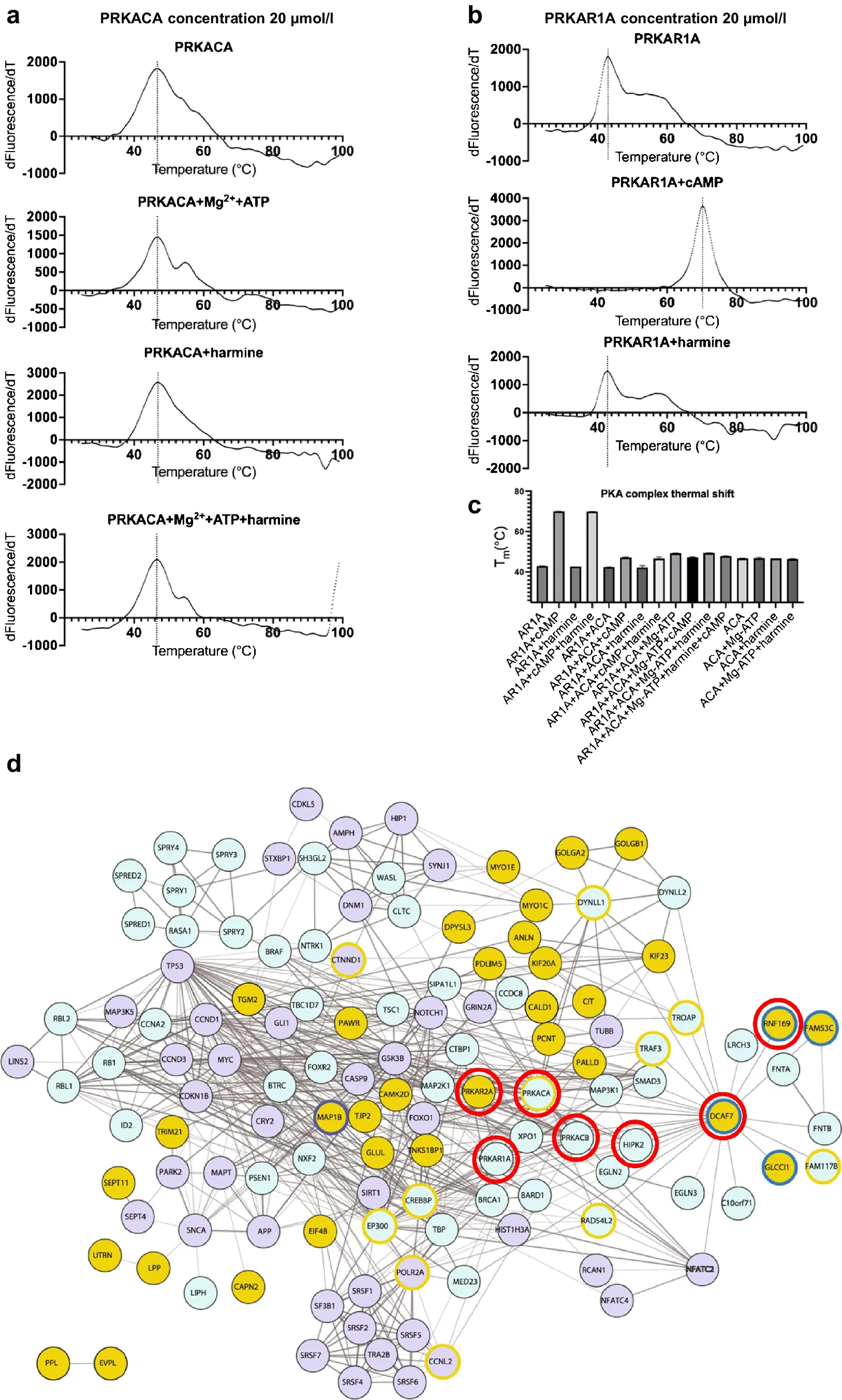
cAMP, but not harmine, binds directly to PRKAR1A and the harmine–DYRK1A interactome. (**a**) In a thermal shift assay, no thermal shift was observed when recombinant human PRKACA was assayed in the presence of ATP and harmine, suggesting that harmine, as expected, does not bind to PRKACA. (**b**) As expected, cAMP caused a dramatic thermal shift in PRKAR1A, its well known target, when recombinant human PRKAR1A was assayed in the presence of cAMP and harmine. In contrast, harmine had no effect on PRKAR1A’s thermal stability, suggesting that there is no direct interaction between harmine and PRKAR1A, at least in vitro and outside of the cellular context. (**c**) A summary of multiple thermal shift experiments. dFluorescence/dT denotes the first derivative of fluorescence intensity with respect to temperature; T_m_ denotes the protein melting temperature derived from the thermal denaturation curves. (**d**) An illustration of the DYRK1A interactome and, by extension, the harmine interactome, adapted with permission from Roewenstrunk, de la Luna and colleagues, under the terms of a Creative Commons Attribution 4.0 International License (http://creativecommons.org/licenses/by/4.0/) [38]. Many of the molecules in this cluster highlighted with red circles in the interactome, including DCAF7, RNF169, PRKACA, PRKACB, PRKAR1A, PRKAR2A, were observed in our own pulldown data (Fig. 6b) and in publicly available datasets such as BioGrid and String. Yellow circles indicate proteins identified in public interaction databases; light blue circles indicate proteins identified in the published DYRK1A interactome. Definitions of molecule abbreviations are provided in ESM Table 2. Multiple members of the DREAM complex (LIN52, RB1, RBL1, RBL2, multiple cyclins and Myc) [21], are present on the left side of the interactome. This leads us to predict that harmine may interact directly with molecules such as these, and links harmine to the identity-enhancing effects in human islets

In the conventional model of PKA shown in Fig. 6c, cAMP interacts with the inhibitory PRKAR1A subunit, resulting in dissociation of PRKAR1A from PRKACA, thereby liberating the catalytic unit to phosphorylate downstream targets such as the CREB superfamily. We reasoned that since harmine and ATP both dock in the same ATP site in DYRK1A [15, 22, 23], and since the structures of cAMP, ATP and harmine share some degree of similarity, it might be possible that harmine could bind directly to PRKAR1A, in a manner similar to cAMP, thereby releasing PRKACA catalytic subunits from PRKAR1A suppression. To test this directly, we used thermal shift assays with recombinant PRKAR1A and PRKACA to determine if either was able to bind to harmine. As shown in Fig. 8a, recombinant PRKACA had a melting point of approximately 47°C, and this was not affected by harmine, indicating that harmine does not bind to isolated recombinant PRKACA. Figure 8b demonstrates that pure recombinant PRKAR1A had a melting point of 43°C, and this was dramatically shifted upward to 70°C in the presence of cAMP, the positive control. In marked contrast, incubation of recombinant PRKAR1A with harmine did not alter its melting point, which remained at 43°C. In aggregate (Fig. 8c), these findings suggest that although harmine clearly influences PKA activity, it likely does not do so through direct interaction with PRKAR1A.

## Discussion

These studies show that members of the DYRK1A inhibitor class of small molecules (Fig. 1) have two important but distinct effects on human beta cells. First, all DYRK1A small-molecule inhibitors induce human beta cells to proliferate [16–28]. This is a central therapeutic property of the DYRK1A inhibitor class, can be mimicked by DYRK1A silencing [15, 17, 24], and results directly from inhibition of the DYRK1A kinase. Second, a small subset of DYRK1A inhibitors (harmine, 2-2c and 5-IT) has an additional beneficial therapeutic effect: unlike the other DYRK1A inhibitors shown in Fig. 1, these compounds also enhance abundance of human beta cell transcription factors, identity markers and function in vitro and in vivo, defining this subgroup as a rare dual-action class within the broader DYRK1A inhibitor family. These properties suggest that some DYRK1A inhibitors may be more effective than others for the treatment of diabetes. Importantly, the beta cell identity-enhancing effects of harmine, 2-2c and 5-IT appear to be independent of DYRK1A, since silencing the entire DYRK family in human islets had no effect on human beta cell identity or phenotypic marker expression (Fig. 5a, b, d). Collectively, these findings demonstrate the following: (1) there is an as-yet-unidentified ‘Target 2’ for harmine, 2-2c and 5-IT, independent of DYRK1A (‘Target 1’), through which this group enhances human beta cell identity or phenotypic marker expression; and (2) INDY, leucettine and other non-beta cell-enhancing DYRK1A inhibitors apparently fail to interact with this putative second ‘Target 2’. An unbiased proteomic screen identified 27 new ‘Target 2’ candidates (Fig. 6b) among which PRKAR1A emerged as the lead. Thermal shift assays using purified recombinant PRKAR1A and PRKACA failed to show direct harmine interactions, suggesting that harmine may act on another unknown ‘Target 2’ protein that modulates PKA activity, or that harmine may interact with PRKAR1A only in the native cellular context.

There is a well-characterised ‘greater DYRK1A-associated interactome’ that includes PRKAR1A and other PKA family members, as described by Rowenstrunk et al, Li et al, and Litovchick and colleagues [38–40] (Fig. 8d), and delineated in protein databases such as BioGrid (https://thebiogrid.org) and String (https://string-db.org). Consistent with this model, our pulldown experiments identified DCAF7 and RNF169 as the highest affinity binders of harmine (Fig. 6b). The ‘DYRK1A interactome’ also includes CREB pathway signalling coregulators EP300 and CBP/CREBBP [41–43] (Fig. 8d). Collectively, these reports suggest that harmine activates PKA signalling and drives human beta cell identity markers through an as-yet-unidentified ‘Target 2’ that may be a member of the ‘DYRK1A interactome’. Additional studies will be required to elucidate which, if any, of the proteins is a direct target of harmine, 2-2c and 5-IT. Finally, it is important to emphasise that DYRK1A itself is unlikely to mediate the human beta cell identity marker effect, since silencing the DYRK family does not block harmine-induced beta cell identity markers in human islets (Fig. 5a, b, d).

We were surprised to observe that harmine activated PKA signalling, because GLP1RAs, which also act in part through PKA pathways, do not stimulate adult human beta cell proliferation [41], though they can stimulate proliferation in juvenile human beta cells [44]. We had shown previously that GLP1 has little or no effect on human beta cell identity markers [16]. On the other hand, in rodent models, studies have suggested that activation of the cAMP–PRKAR1A–CREB–CBP pathway can promote beta cell proliferation or insulin secretion [45–48]. Montminy and colleagues suggested that CREB can recruit the coactivator EP300 to promoters or enhancers of the beta cell transcription factor, NeuroD1 [41]. Other studies have suggested that PKA can influence beta cell identity marker expression through histone methylation [42] or MafA induction [43]. In a rare human islet study, Tiemann et al suggested that cAMP–PKA–CREB signalling represses the alpha cell transcription factor ARX1 [49]. However, no prior studies have reported PKA induction of human beta cell identity markers. With regard to timing of these events, our studies show that harmine increases PKA activity (^133^Ser-CREB1 phosphorylation) within minutes (Fig. 6d–f and ESM Fig. 4), that subsequent induction of *PDX1*, *MAFA* and *NKX6.1* gene expression is rapid (within 2–4 h of harmine exposure; ESM Fig. 2) and that PKA activation is required for induction of MafA and PDX1, since it is inhibited by the PKA inhibitor H89 (Fig. 7d).

PKA is a complex molecule, as are its multiple subunits and subcellular locations. Lu et al recently described a 3D structure of the PKA heterotetramer [37], suggesting that cAMP may induce structural rearrangements of the holoenzyme rather than simple subunit dissociation. Such mechanisms would not be detected in experiments using isolated recombinant proteins, as in our thermal shift assays. Further, PKA signalling is spatially complex, with PRKAR1A reported to form liquid–liquid phase separated condensates that may serve as local cAMP reservoirs, localised to multiple subcellular domains, through interactions with AKAP scaffold proteins [50]. Where precisely harmine acts within this spatially organised PKA signalling network remains unknown. Moreover, which of the many CREB superfamily transcription factors (CREBs, ATFs and others) are relevant, how they modulate beta cell identity markers, which specific genes and/or enhancers they target in the beta cell epigenome, which co-activators (e.g. EP300, CBP, CRTC, etc.) might be required, and which (and how) these might activate specific identity markers will provide fruitful areas for future research.

### Limitations

This study has limitations and raises several important questions. First, we have not definitively identified ‘Target 2’ for harmine, although we suggest it interacts with or regulates PKA. Second, we have not demonstrated how ‘Target 2’ mediates the beta cell identity-enhancing effects of harmine. Understanding this mechanism will require extensive studies including and excluding multiple candidate CREB/ATF superfamily members and genome-wide (ChIPseq, CUT&RUN) studies to identify relevant promoters, enhancers and target genes. Third, we have not shown how harmine interacts with the broader DYRK1A interactome (Fig. 8d), and the roles of DCAF7, RNF169 and other DYRK1A-associated proteins in human beta cells have not yet been explored. Fourth, if harmine activates PKA to enhance human beta cell identity markers, why does GLP1, which also acts via PKA, fail to activate human beta cell identity marker expression [18]? The PKA structural model shown in Fig. 6c will continue to evolve over time [37, 51] and may shed light on this question. One possibility is that GLP1R–PKA signalling occurs in different intracellular subcompartments not dissected in these studies. Another possibility is that harmine may activate PKA more strongly or through distinct regulatory mechanisms, as compared with GLP1. Finally, these studies do not address whether harmine and related compounds will be safe or effective in humans with diabetes. Encouragingly, single-dose studies in humans show that harmine is well tolerated [20], and 3 months treatment in mice revealed no abnormal histological or proliferation effects in a panel of 11 relevant target tissues [19]. The human safety questions will need to be addressed going forward in complete preclinical safety and toxicology studies. In addition, although human islets from both male and female non-diabetic donors were included in this study, individual experiments involved relatively small numbers of donor preparations and were designed to evaluate treatment effects using donor-matched comparisons rather than biological sex as an experimental variable. Consequently, the study was not powered to detect sex-specific differences in the response to harmine or PKA activation. Future studies with larger, balanced donor cohorts will be needed to determine whether biological sex influences the magnitude or mechanism of these responses and to establish the generalisability of these findings across sexes.

## Supplementary Information

Below is the link to the electronic supplementary material.

Supplementary file1 (PDF 1454 KB)

Supplementary file2 (XLSX 3592 KB)

## Data Availability

The datasets generated and/or analysed during the current study are available from the corresponding author upon reasonable request. Data will be made available upon publication of this article and will remain available for at least 5 years following publication. Data will be shared with qualified researchers for academic, non-commercial research purposes, subject to institutional approvals, material transfer agreements where applicable, and compliance with relevant ethical and data-sharing regulations.

## Abbreviations

CLK: CDC-like kinase
DYRK: Dual tyrosine-regulated kinase
GSIS: Glucose-stimulated insulin secretion
5-IT: 5-Iodotubercidin
MED21: Mediator 21
PKA: Protein kinase A

## Authors’ relationships and activities

PW, AFS, KK, RJD and AGO are inventors on patents filed by The Icahn School of Medicine at Mount Sinai. PaulexBio has licensed this patent portfolio from The Icahn School of Medicine at Mount Sinai. AFS, RJD and AGO are consultants for PaulexBio. AGO consults for Sun Pharmaceuticals. The other authors declare that there are no relationships or activities that might bias, or be perceived to bias, their work.
