# Supplementary material for: Harmine selectively enhances human beta cell identity markers and function via protein kinase A pathways
Source: Diabetologia. 2026 Sep 21;69(12):3592–608. doi: 10.1007/s00125-026-06857-5 (PMC13714090; doi:10.1007/s00125-026-06857-5)
Supplement: Supplementary file 1 [file 125_2026_6857_MOESM1_ESM.pdf]

Electronic Supplementary Material (ESM))

Human islets

Isolated, de-identified human pancreatic islets from non-diabetic organ donors were obtained through the NIH Integrated Islet Distribution Program (IIDP), Prodo Laboratories (Irvine, CA, USA), the Alberta Diabetes Institute (Edmonton, AB, Canada), and the Transplant Surgery Department at the University of Chicago (Chicago, IL, USA). Donor demographic and islet preparation characteristics are provided in ESM human islet checklist.

Human islets were isolated from cadaveric donor pancreata using standard collagenase digestion and purification procedures performed at the respective isolation centers. All islet preparations were obtained from de-identified donors and were exempt from Institutional Review Board approval. Upon receipt, islets were cultured in CMRL-1066 medium supplemented with 10% fetal bovine serum, 1% penicillin-streptomycin, and 2 mmol/l glutamine at 37°C in a humidified atmosphere containing 5% CO_2. Islets were allowed to recover overnight prior to experimental use unless otherwise indicated.

A total of 91 independent human islet preparations were used in this study. Donor age, sex, BMI, HbA1c, purity, viability, and source information are summarized in ESM human islet checklist. Human islets from both male and female donors were included where available. Because individual experiments involved relatively small numbers of donor preparations, analyses were not stratified by donor sex.

Chemicals

Accutase was purchased from Mediatech (Manassas, VA, USA; Cat. No. 25-058-CI). Harmine was purchased from Sigma-Aldrich (St. Louis, MO, USA; Cat. No. 286044). Leucettine-41 was purchased from AdipoGen (San Diego, CA, USA; Cat. No. AG-MR-C0023). INDY (Cat. No. 4997) and 5-iodotubercidin (5-IT; Cat. No. 1740) were purchased from Tocris Bioscience (Bristol, UK). 8-CPT-cAMP, 6-Bnz-cAMP, dcAMP, GNF4877, CC-401 and H89 were purchased from MedChemExpress (Monmouth Junction, NJ, USA). Forskolin was purchased from Selleck Chemicals (Houston, TX, USA; Cat. No. S2449). Compound 2-2c was synthesized at the Drug Discovery Institute and the Department of Pharmacological Sciences at the Icahn School of Medicine at Mount Sinai as previously described [23].

Stock solutions of harmine, leucettine-41, INDY, 5-IT, GNF4877, CC-401, forskolin and H89 were prepared in DMSO and stored according to the manufacturers' recommendations. Vehicle controls contained equivalent concentrations of DMSO.

Adenoviruses and Transduction: For adenoviral silencing, four target shRNA sequences for each target gene were designed using the Thermo Fisher RNAidesigner online tool. Target sequences were cloned into Block-iT U6 RNAi entry vector (K494500, Invitrogen), and silencing efficiency was evaluated in HEK293 cells. The most effective target sequences were used to generate Ad-shRNAs in the Block-iT adenoviral RNAi vector (K494100, Invitrogen). The virus that silences both DYRK1A and DYRK1B simultaneously has been reported previously [15, 17]. Adenoviruses for silencing RBL2/p130/RBL1/P107/Rb1, E2F4/E2F5, and CLK1/CLK2/CLK4 have been reported previously [21]. Ad.NFATC1 has been previously described [25]. Mouse Ad.ca-nfatc1 and ca-nfatc2 were gifts from Drs. Alan Attie and Mark Keller, Department of Biochemistry, University of Wisconsin. NFAT4 (NFATC3), CA-NFAT2 (CA-NFATC1), CA-NFAT3 (CA-NFATC4) are described in detail in reference [21]. Ad-shRNA-PRKAR1A was prepared using U6 RNAi entry vector (45-0511, Invitrogen). The PRKAR1A shRNA target sequence is GGGATAACTTCTATGTGATTG. The Ad-V5-PRKACA construct was generated by PCR amplifying PRKACA from the Addgene pDONR223-PRKACA plasmid (Cat. #82311), using a forward primer containing an N-terminal V5 epitope tag. The tagged PCR product was cloned into a Gateway destination vector under the control of the RIP promoter.

Human Islet Dispersion and Virus Infection. Islets were centrifuged at 1500 rpm for 5 min, washed twice in phosphate-buffered saline (PBS), re-suspended in 1 ml of Accutase and incubated for 10 min at 37°C. During digestion, the islets were dispersed by gentle pipetting up and down every 5 min for 10 sec. Complete RPMI medium containing 11 mmol/L glucose, 1% penicillin/streptomycin with 10% fetal bovine serum (FBS) was then added to stop the digestion. Dispersed cells were then centrifuged for 5 min at 1500rpm, the supernatants removed, the pellets re-suspended in complete medium, and the cells then plated on coverslips with 30 µl of cell suspension per coverslip. Poly-D-Lysine/Laminin-treated cover slides or chamber slides were used. Cells were allowed to attach for 2 hr at 37°C or were transduced with adenovirus for 2 hr. After 2 hr, 500 µl complete RPMI medium was added in each well to terminate adenoviral transduction. Cells were cultured for 24-96 hr as described in the Figure Legends.

Compound Treatments. For compound treatments, dispersed islet cells were allowed to recover on coverslips for 24 hr. RPMI complete medium was then replaced with fresh medium containing harmine or vehicle (0.1%

DMSO) for 72-96 hr. Doses used were derived from publications that demonstrated proliferation in human beta cells[15, 17, 21-23, 27, 29, 30], and were harmine (10 μmol/l), 5-IT (1 μmol/l), GNF4877 (2 μmol/l), INDY (15 μmol/l), Leucettine-41 (20 μmol/l), CC-401 (10 μmol/l), Forskolin (10 μmol/l) and H89 (15 μmol/l). For the combination treatment with adenovirus and compounds, islet cells were transduced first, and then 24 hours later the cells were treated with compound.

qPCR Methods. RNA was isolated and quantitative RT-PCR was performed as described previously [3]. Gene expression in dispersed islets was analyzed by real-time QuantStudio Real-Time PCR System. Primer sequences are listed in **ESM Table 1**.

Immunoblotting and Immunocytochemistry. Immunoblotting procedures. Immunoblots were performed on whole human islets as described previously [15–18, 21]. Primary antibodies were: anti-PDX1 (Abcam, Cambridge, UK; Cat. No. ab219207; RRID:AB_2890673), anti-MAFA (Abcam, Cambridge, UK; Cat. No. ab264418; RRID:AB_2889168), anti-NKX6.1 (Developmental Studies Hybridoma Bank, Iowa City, IA, USA; clone F55A10-c; RRID:AB_531793), anti-V5 (Invitrogen, Carlsbad, CA, USA; Cat. No. 46-0705; RRID:AB_2556564), anti-PRKAR1A (Invitrogen, Carlsbad, CA, USA; Cat. No. MA5-24981; RRID:AB_2725046), anti-CREB (Proteintech, Rosemont, IL, USA; Cat. No. 67927-1-Ig; RRID:AB_2881438), anti-phospho-CREB (Ser133) (Cell Signaling Technology, Danvers, MA, USA; Cat. No. 9198; RRID:AB_2561044), and anti-GAPDH (Cell Signaling Technology, Danvers, MA, USA; Cat. No. 2118; RRID:AB_561053).

Immunoblot Quantification. Western blot bands were quantified using ImageJ (NIH). Blot images were converted to 8-bit and analyzed without altering contrast. Identical rectangular areas were drawn around each band, and integrated density was measured. Background intensity was taken from a nearby region and subtracted from each band. Target protein signals were normalized to their corresponding loading control and then expressed relative to the control condition, which was set to 1.0.

Immunocytochemistry and Antisera. Immunocytochemistry was performed on coverslips fixed in freshly prepared 4% paraformaldehyde. Accutase-dispersed human islet cells were plated on coverslips as previously described [35]. Primary antibodies were: anti-C-peptide (Developmental Studies Hybridoma Bank, Iowa City, IA, USA; clone GN-ID4; RRID:AB_531896), anti-PDX1 (Abcam, Cambridge, UK; Cat. No. ab47308; RRID:AB_2162359), anti-NKX6.1 (Developmental Studies Hybridoma Bank, Iowa City, IA, USA; clone F55A10-c; RRID:AB_531793), anti-ENTPD3 (kindly provided by Dr Alvin Powers, Vanderbilt University Medical Center, Nashville, TN, USA), and anti-phospho-CREB (Ser133) (Cell Signaling Technology, Danvers, MA, USA; Cat. No. 9198; RRID:AB_2561044).

Fluorescence Image Quantification. Fluorescence staining was quantified using ImageJ (NIH). Images were converted to 8-bit and analyzed using identical settings across all samples. A fixed-size region of interest (ROI) was drawn around each cell or area of interest, and integrated fluorescence intensity was measured. Background fluorescence from an unstained adjacent region was subtracted from each measurement. Fluorescence values were then normalized to the control group and expressed relative to control.

Human Islet Proteomics and Pulldown Assays. For the studies on the differential protein response to harmine, human islets in **Fig. 4**, three different human islet preparations were treated for 72 hours with vehicle (0.1% DMSO) vs. harmine (10 μmol/l), then lysed using lysis buffer containing 200 mM EPPS (pH 8.5) and 8 M urea, with complete protease and PhosSTOP phosphatase inhibitors, then flash-frozen and shipped to the Proteomics Facility at Harvard Medical School Center for Mass Spectrometry. There, samples were subjected to the facility's standardized workflow. Peptides were purified and analyzed by liquid chromatography–tandem mass spectrometry (LC-MS/MS). Raw spectra were processed with the facility's established pipeline, including peptide identification against the human UniProt database and label-free quantification. Protein-level abundances were generated after peptide-spectrum matching, false-discovery rate control (1% FDR at peptide and protein levels), and normalization using the Center's standard procedures. The raw protein expression matrix was then log2-transformed and median-centered prior to analysis. The dataset included nine donor pairs, each consisting of three samples processed across different batches. Differential protein expression was assessed on 7,687 proteins detected in at least two of the three batches. LIMMA models were fitted using a design matrix including treatment condition (Harmine vs DMSO) and donor pair ID. Batch correction was not applied because each batch was perfectly confounded with donor identity, and including both would make the design matrix singular; the paired model fully accounts for between-pair differences. The Harmine–vs–DMSO contrast was estimated using empirical Bayes moderation, and the full DPE table was extracted with FDR correction.

For the pulldown studies (**Fig. 6a-b**), harmine was purchased from (Sigma, St. Louis). 2-2c and compound 15 were synthesized at Mount Sinai as described [22, 23]. Pulldown of human islet lysates, magnetic bead coupling to compound 15 and graded elution, followed by shotgun proteomics were performed at Evotec International GmbH, Munich, Germany.

Protein Purification For Thermal Shift Assay. A plasmid encoding human PRKAR1A plasmid was a gift from William Hahn & David Root (Addgene plasmid # 23741; http://n2t.net/addgene:23741; RRID: Addgene_23741). The plasmid used to express PRKACA in mammalian cells was used as a template for bacterial expression. We subcloned both the regulatory (PRKAR1A) and catalytic subunit (PRKACA) separately into His-Sumo N-terminal tagged vectors. For expression, the plasmids were introduced into LOBSTR cells, a modified BL21 (DE3) strain. For PRKACA, the plasmid was co-transformed with lambda phosphatase (in a pCDF vector) to reduce toxicity. Terrific Broth (TB) media supplemented with kanamycin for PRKAR1A and both kanamycin and streptomycin for PRKACA at 50 μg/ml final concentration was inoculated with 1:1000 dilution of an overnight culture. The culture was grown at 37ºC and the temperature was reduced to 16ºC once the OD_{600} reached 1.5. Protein production was induced overnight by adding IPTG to a final concentration of 0.4 mmol/l at 16ºC. The cells were harvested the next day at 4ºC and then resuspended in TBS (20 mmol/l Tris pH 8.0 and 250 mM NaCl, supplemented with 1 mM PMSF and 0.1 mg/ml Lysozyme). The cells were then lysed by sonication. The supernatant was supplemented with 40 mmol/l imidazole and then purified by IMAC. After batch binding, the column was washed with TBS containing 50 mM imidazole and then eluted with TBS containing 250 mM Imidazole and 10% glycerol. After cleaving the His-Sumo tag with Sumo protease overnight, the protein was further purified through Superdex-200 gel filtration sizing in TBS containing 20 mmol/l Tris pH 8.0 and 150 mmol/l NaCl.

Protein Thermal Shift Assay for PRKAR1A and PRKACA. The thermal shift assay to assess the harmine and cAMP binding to PRKAR1A and PRKACA, individually and as a complex was performed on a QuantStudio 3 real-time PCR system (Applied Biosystems). In the final reaction volume of 20 mL, proteins were diluted to a final concentration of 0.5 mg/ml, in TBS buffer. Both cAMP and Mg-ATP in water were diluted to a final 1 mM concentration. Harmine was added to a final concentration of 0.1 mM at 1% DMSO from 1 mM in 10 % DMSO stock. 10x SYPRO orange (Applied Biosystems) was prepared by adding 0.5 ml of 5000x to 250 mL of TBS buffer and in the final reaction mix, 2 ml of this dye was added. The reactions were kept at 25°C for 2 min and then heated from 25°C to 95°C with a rate of 0.05°C/S. The change in the fluorescence intensities of SYPRO orange was monitored as a function of the temperature and analyzed by Protein Thermal Shift Software 1.3. Each reaction was performed in triplicate.

Statistics. All statistical analyses were performed using GraphPad Prism (version X). Because human islets from different donors exhibit substantial inter-donor variability, all analyses were conducted using donor-matched approaches, with each donor serving as an independent biological replicate. For qPCR experiments, raw Ct values were first normalized to an internal reference gene to obtain ΔCt values. ΔΔCt values were then calculated by normalizing to the control condition (DMSO). Statistical analyses were performed on ΔCt values rather than normalized fold-change values to preserve variance and meet assumptions of statistical testing, while fold-change values ($2^{-\Delta\Delta Ct}$) were used for data presentation. Data distribution was assessed for normality using the Shapiro–Wilk test. For comparisons between two matched groups, paired two-tailed Student's t-tests were used for normally distributed data, whereas Wilcoxon signed-rank tests were applied when normality could not be assumed. For comparisons involving more than two matched groups, repeated-measures one-way ANOVA was used for normally distributed data, followed by Dunnett's multiple-comparisons test to compare each treatment group with the control condition. When normality assumptions were not met, non-parametric analyses were performed using the Friedman test followed by Dunn's multiple-comparisons test. Data are presented as mean ± SEM. A value of $p<0.05$ was considered statistically significant.

ESM Table 1. PCR Primers

Cyclophilin A	CACCGTGTTCTTCGACATTG	TGAAGTCACCACCCTGACAC
INS	TCACACCTGGTGGAAGCTCTCTA	ACAATGCCACGCTTCTGCAGGGAC
MAFA	GAGCGGCTACCAGCATCAC	CTCTGGAGTTGGCACTTCTCG
NKX6.1	ACACGAGACCCACTTTTTCCG	TGCTGGACTTGTGCTTCTTCAAC

PAX4	AGTCCTGCGGGCATTACAG	GGGAGAAGATAGTCCGATTCCG
PCSK1	GGACCTCTGAGTATGACCCG	AGCTTTGGCATTTAGCAAGCC
MAFB	TCAAGTTCGACGTGAAGAAGG	GTTCATCTGCTGGTAGTTGCT
NeuroD1	GTCTCCTTCGTTCAGACGCTT	AAAGTCCGAGGATTGAGTTGC
NGN3	CTAAGAGCGAGTTGGCACTGA	GAGGTTGTGCATTCGATTGCG
SLC2A2 (GLUT2)	GCTGCTCAACTAATCACCATGC	TGGTCCCAATTTTGAAAACCCC
PCSK2	GGGAAAGGTGTTACCATTGGAA	CCAGTCATCTGTGTACCGAGG
SLC2A1 (GLUT1)	GGCCAAGAGTGTGCTAAAGAA	ACAGCGTTGATGCCAGACAG
TXNIP	ATATGGGTGTGTAGACTACTGGG	GACATCCACCAGATCCACTACT
HIPK2	AATAGAGCCGAGTTCCAACTGG	GTCTGCTCGTAAGGTAGGCTT
ALDH1A3	TGAATGGCACGAATCCAAGAG	CACGTCGGGCTTATCTCCT
GLP1R	GGTGCAGAAATGGCGAGAATA	CCGGTTGCAGAACAAGTCTGT
NKX2.2	CCGGGCCGAGAAAGGTATG	GTTTGCCGTCCCTGACCAA
PCSK1	GGACCTCTGAGTATGACCCG	AGCTTTGGCATTTAGCAAGCC
PAX4	CTACCGCACAGGTGTCTTGG	CGTTGGATTTCCCAGGCAA
UCN3	CCCACAAGTTCTACAAAGCCA	TCCCGAAGAGGCGTCTCTG
SIX2	AAGGCACACTACATCGAGGC	AAGGCACACTACATCGAGGC
SIX3	CAAGGAGTCTCACGGCAAG	GCAATGCGTCTTCTGCTCG
ENTPD3	GACTTTAGGGGTGCTCAAATCA	CATGTGCCACAGGTTCTTCTC
SLC30A8	ATCGAAGCCTCCCTCTAAGC	CACATGCCAGGTACACTAGCA
CHGA	TAAAGGGGATACCGAGGTGATG	TCGGAGTGTCTCAAAACATTCC
CHGB	CGAGGGGAAGATAGCAGTGAA	CAGCATGTGTTTCCGATCTGG
PRKAR1A	CACTGCTCGACCTGAGAGAC	CCGCATCTTCCTCCGTGTAG

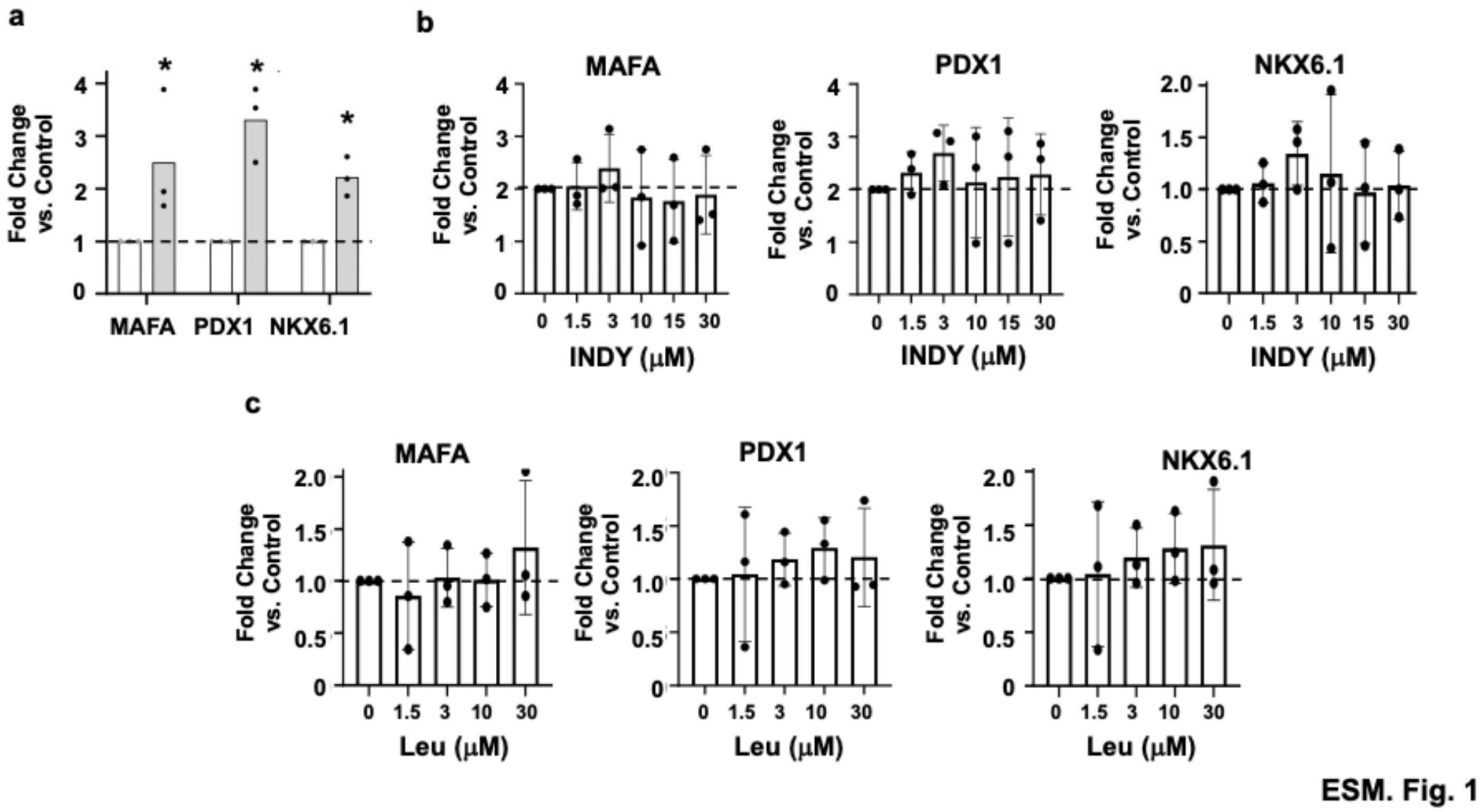

ESM Fig. 1. Dose-Response Effects For Harmine, INDY and Leucettine on MAFA, PDX1 and NKX6.1. a. Human islets were treated with harmine (10 μmol/L) for 24 h, and expression of β-cell identity genes was analyzed by RT-qPCR. Harmine increased expression of MAFA, PDX1 and NKX6.1 compared with DMSO-treated controls. Open bars indicate vehicle (0.1% DMSO) and grey bars indicate harmine at 10 μmol/L. **b.** Human islets were treated with increasing concentrations of INDY for 24 h, followed by RT-qPCR analysis of MAFA, PDX1, and NKX6.1 expression. High dose INDY treatment induced no significant effect on β-cell identity gene expression. **c.** Human islets were treated with increasing concentrations of leucettine for 24 h, followed by RT-qPCR analysis of MAFA, PDX1 and NKX6.1 expression. High dose leucettine treatment induced no significant effects on β-cell identity gene expression. Gene expression was normalized to the housekeeping gene cyclophilin, and calculated using the ΔΔCt method. Data are presented as fold-change relative to 0.1% DMSO-treated controls, indicated as “0”. Statistical analyses were performed using ΔCt values prior to fold-change transformation. For comparisons between DMSO and harmine treatment groups, Student’s paired t-test was used. For INDY and Leucettine-41 dose-response experiments, statistical analyses were performed using one-way ANOVA followed by Dunnett’s multiple comparisons test. Data are shown as mean ± SEM from independent human islet donors. *P < 0.05 versus DMSO.

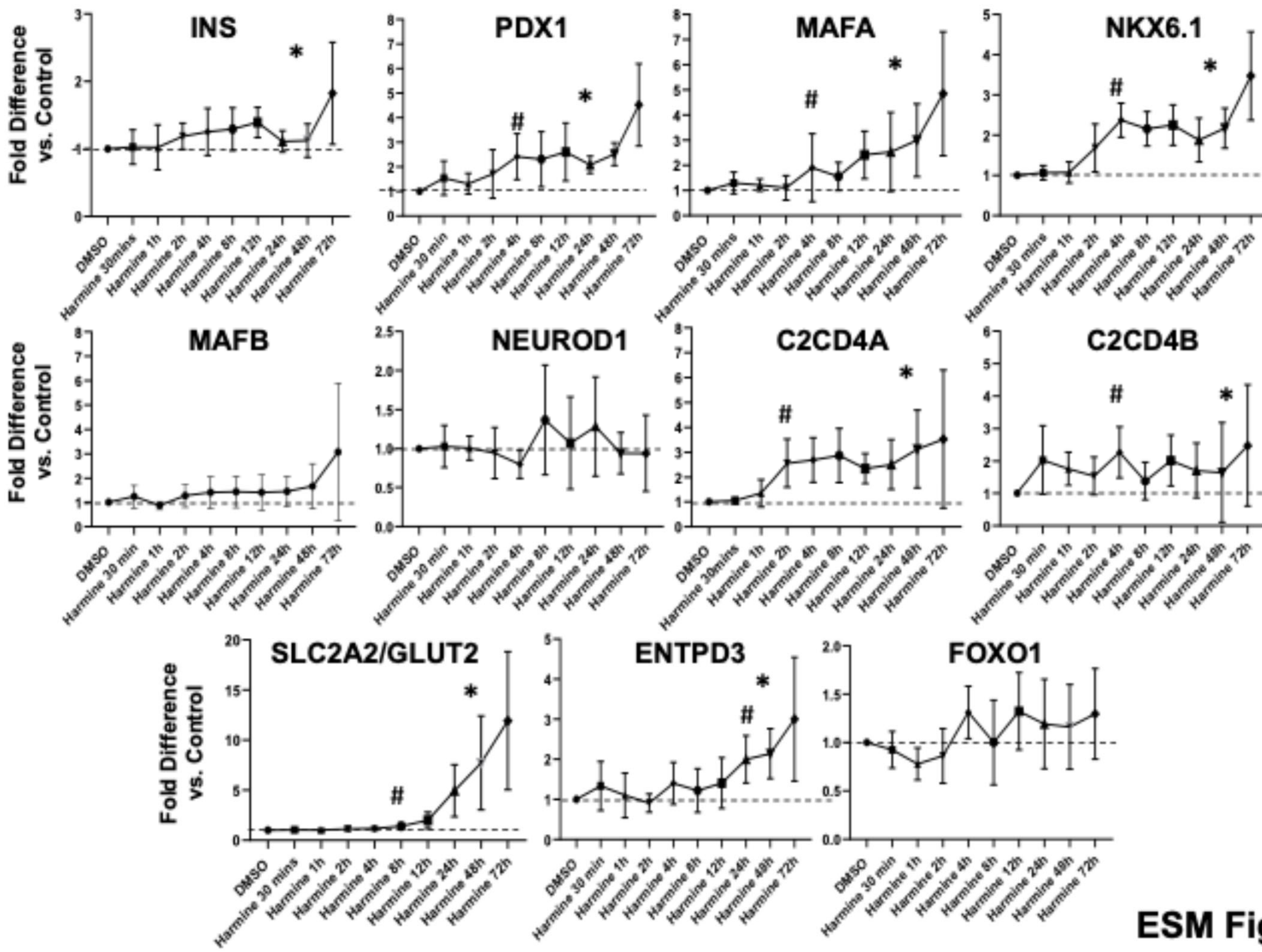

ESM Fig. 2

ESM Fig. 2. Time Course of Increase in Selected Human Beta Cell Identity Markers. Note that PDX1, MAFA, NKX6.1 and C2CD4A rise within 2-8 hours, whereas MAFB, ENTPD3 and SLC2A2 (GLUT2) require 12-24 hours to respond. Note also that other transcription factors (NEUROD1, FOXO1, C2CD4B) show little response. Note that Insulin mRNA changes only modestly, as reported previously, although insulin secretion increased *in vitro* and *in vivo* in response to harmine[16,19]. Asterisks represent P<0.05 for the complete dataset vs. time 0 by one-way ANOVA, and the # symbols indicate the earliest time point at which the factor in question becomes elevated, as determined by paired T-tests vs. time 0. Fold-change values relative to control are shown for presentation; statistical analyses were performed on donor-matched ΔCt values. Asterisks indicate $p < 0.05$ determined by repeated-measures one-way ANOVA across time points, followed by Dunnett's multiple comparison test comparing each time point to time 0. Hash (#) symbols indicate the earliest time point at which a significant increase was observed.

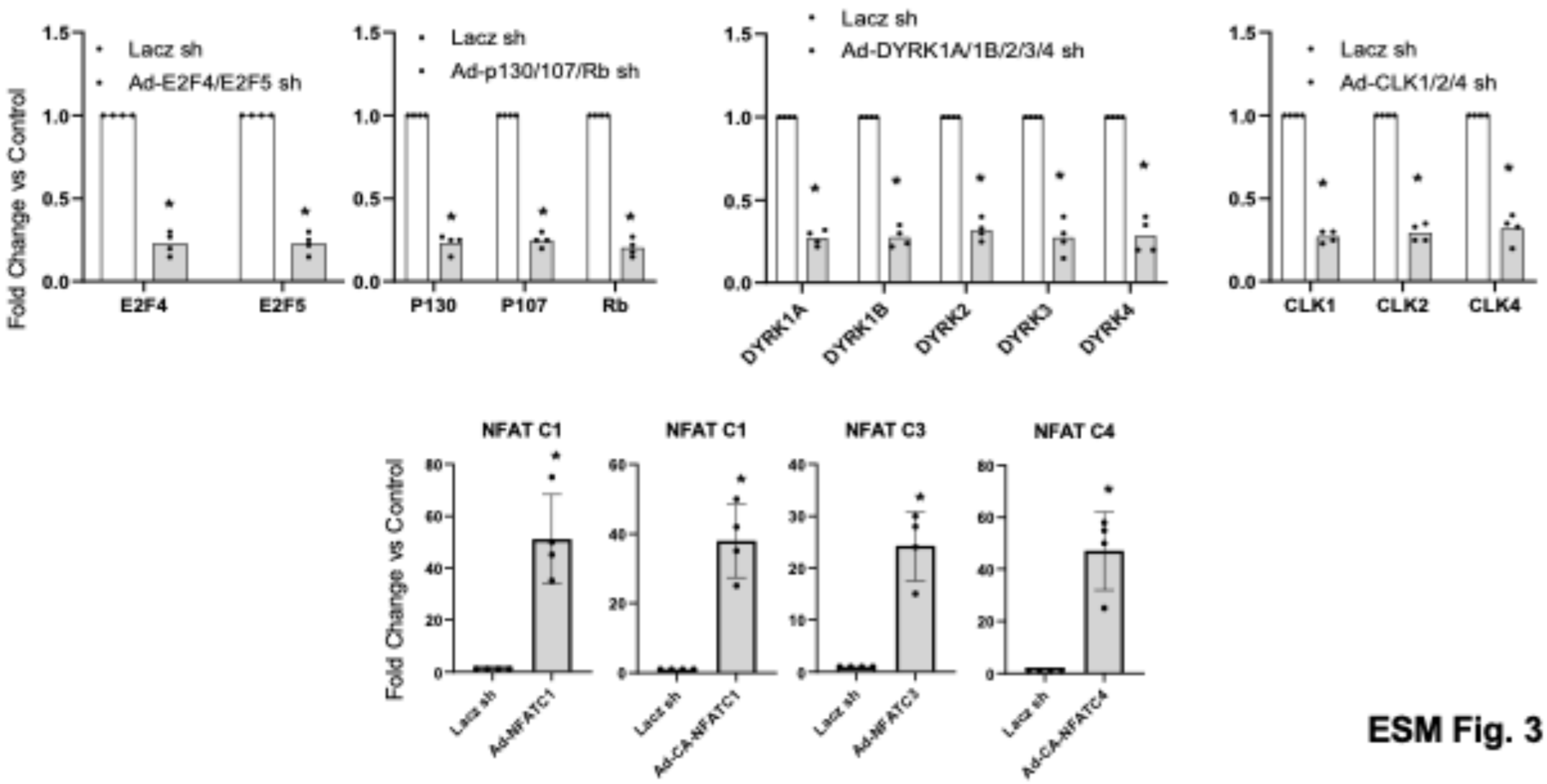

ESM Fig. 3

ESM Fig. 3. Validation of Adenovirus-Mediated Gene Silencing and Overexpression Efficiency in Human Islets. Human islets were transduced with adenoviruses expressing shRNAs targeting components of the DREAM complex (E2F4, E2F5, p130/RBL2, p107/RBL1, RB1), the DYRK family (DYRK1A, DYRK1B, DYRK2, DYRK3, DYRK4), or CDC-like kinases (CLK1, CLK2, CLK4), or with control LacZ shRNA adenovirus. mRNA levels were quantified by RT–qPCR and expressed as fold change relative to control LacZ shRNA–treated islets. Robust and specific knockdown of each target gene was achieved across independent human islet preparations. Bottom panels show adenoviral overexpression of NFAT family members (NFATC1, constitutively active NFATC1, NFATC3, and NFATC4) in human islets, demonstrating efficient overexpression relative to LacZ control virus. Fold-change values relative to control are shown for presentation; statistical analyses were performed on donor-matched ΔCt values. Asterisks indicate $p < 0.05$ determined by repeated-measures one-way ANOVA, followed by Dunnett's multiple comparison test comparing with control. Data are shown as mean ± SEM from n = 4 independent human islet donors, with individual donor values indicated by dots.

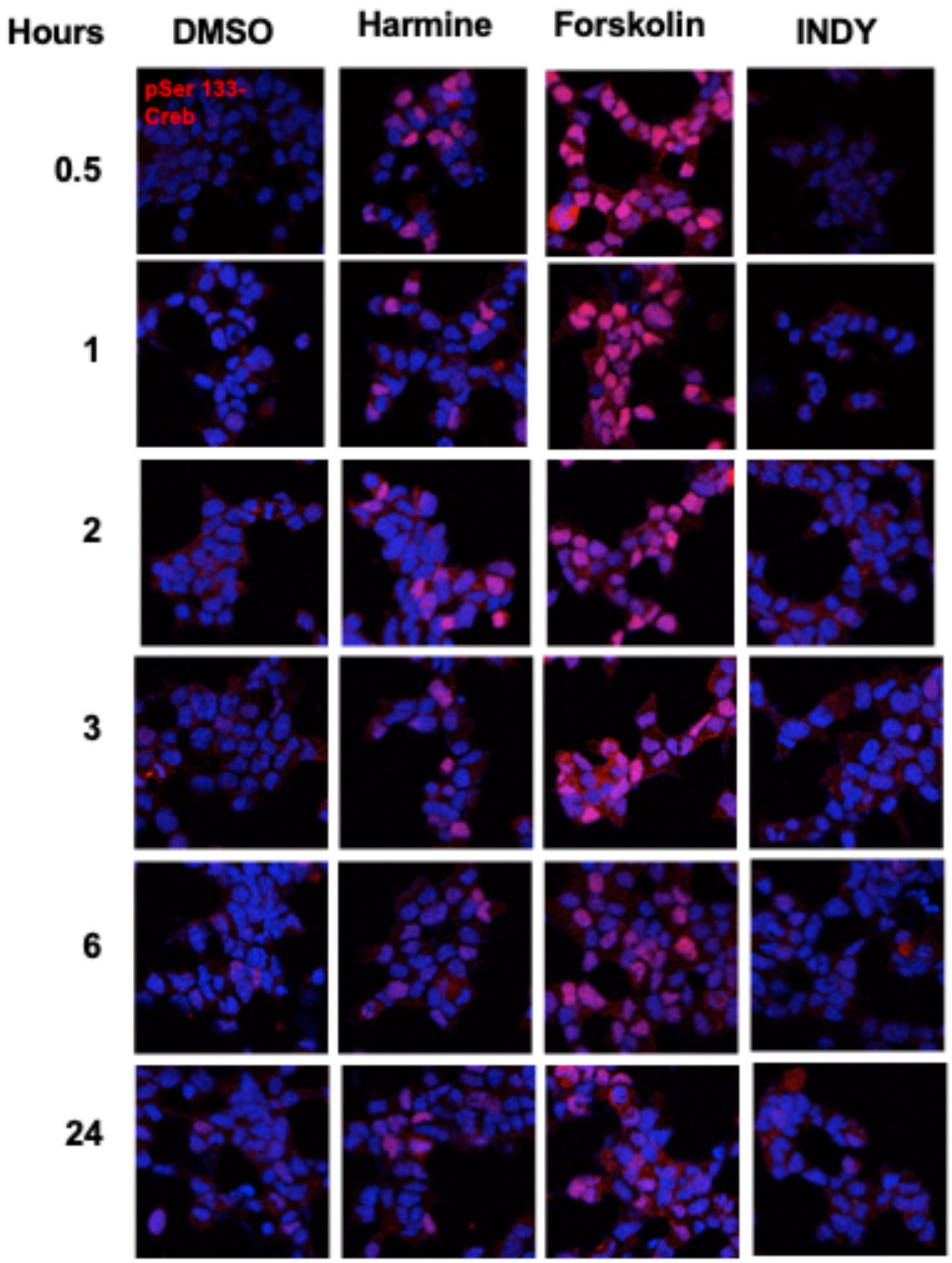

ESM Fig. 4

ESM Fig. 4. Time Course of Harmine and Forskolin Induction of ^{Ser133}P-CREB In Mouse R7T1 Cells. The point is that harmine, like forskolin, induces ^{Ser133}P-CREB phosphorylation in R7T1 cells, beginning within 30 min, and gradually declining over 2-3 hours, paralleling the effects of forskolin.

ESM Checklist for reporting human islet preparations used in research

Adapted from Hart NJ, Powers AC (2018) Progress, challenges, and suggestions for using human islets to understand islet biology and human diabetes. Diabetologia https://doi.org/10.1007/s00125-018-4772-2

[a]If you have used more than eight islet preparations, please complete additional forms as necessary
[b]For example, IIDP, ECIT, Alberta IsletCore
[c]Please specify the therapy/therapies
[d]Time of islet culture at the isolation centre, during shipment and at the receiving laboratory
[e]Please specify the test and the results

**Islet preparation**	**1**	**2**	**3**	**4**	**5**	**6**	**7**	**8[a]**
**MANDATORY INFORMATION**								
Unique identifier	Hu1197	HP20220	H268	Samn13254972	Samn37068473	Samn13149867	Samn37737368	HP23270
Donor age (years)	44	60	24	60	51	28	50	34
Donor sex (M/F)	M	M	F	F	M	M	M	M
Donor BMI (kg/m^2)	28.84	28.3	39.38	44.4	30.4	30.8	38.6	27
Donor HbA_{1c} or other measure of blood glucose control	5.5	5.6	5.7	5.6	6.3	5.2	5.8	5.5
Origin/source of islets[b]	IIDP	Prodo	U of Chicago	IIDP	IIDP	IIDP	IIDP	Prodo
Islet isolation centre	Southern California Islet Cell Resources Center	Prodo	U of Chicago	Wisconsin	Imagine Islet Center	Southern California Islet Cell Resource Center	Southern California Islet Cell Resource Center, City of Hope	Prodo
Donor history of diabetes? Please select yes/no from drop down list	No	No	No	No	No	No	No	No
**If Yes, complete the next two lines if this information is available**								

Diabetes duration (years)								
Glucose-lowering therapy at time of death[c]								
**RECOMMENDED INFORMATION**								
Donor cause of death	Stroke	Stroke	N/A	Head Trauma	Stroke	Head Trauma	Head Trauma	Stroke
Warm ischaemia time (h)								
Cold ischaemia time (h)	7.2	N/A	N/A	6.2	11.9	6	N/A	N/A
Estimated purity (%)	80	85	N/A	99	80	80	85	85
Estimated viability (%)	N/A	95	N/A	99	85	99	95	95
Total culture time (h)[d]								
Glucose-stimulated insulin secretion or other functional measurement[e]								
Handpicked to purity? Please select yes/no from drop down list	No	No	No	No	No	No	No	No
Additional notes								

Islet preparation	9	10	11	12	13	14	15	16
**MANDATORY INFORMATION**								
Unique identifier	Hp23262	HP23258	Samn37350251	HP23254	Hp23200	Samn36471214	HP23189	Samn35848421
Donor age (years)	64	39	43	42	57	40	33	22
Donor sex (M/F)	M	M	F	M	M	M	F	M
Donor BMI (kg/m^2)	23.2	26.9	29.89	26.5	30.8	24.2	28.8	31.79
Donor HbA_{1c} or other measure of blood glucose control	4.8	5.2	5.2	4.8	5.2	5.2	5.5	5.1
Origin/source of islets[b]	Prodo	Prodo	IIDP	Prodo	Prodo	IIDP	Prodo	IIDP
Islet isolation centre	Prodo	Prodo	Wisconsin	Prodo	Prodo	Wisconsin	Prodo	Wisconsin
Donor history of diabetes? Please select yes/no from drop down list	No	No	No	No	No	No	No	No
**If Yes, complete the next two lines if this information is available**								
Diabetes duration (years)								

Glucose-lowering therapy at time of death[c]								
**RECOMMENDED INFORMATION**								
Donor cause of death	Stroke	Stroke	Head trauma	Stroke	Anoxia	Stroke	Anoxia	Anoxia
Warm ischaemia time (h)								
Cold ischaemia time (h)	N/A	N/A	6	N/A	N/A	6	N/A	9
Estimated purity (%)	90	90	88	85	95	73	85	90
Estimated viability (%)	95	95	98	95	95	98	95	98
Total culture time (h)[d]								
Glucose-stimulated insulin secretion or other functional measurement[e]								
Handpicked to purity? Please select yes/no	No	No	No	No	No	No	No	No

from drop down list								
Additional notes								

Islet preparation	17	18	19	20	21	22	23	24
**MANDATORY INFORMATION**								
Unique identifier	Samn35301006	HP23123	Samn34411471	HP23104	Samn34033792	HP23019	Samn32875450	Samn32641506
Donor age (years)	52	47	45	64	39	55	60	16
Donor sex (M/F)	M	M	M	F	M	M	F	M
Donor BMI (kg/m^2)	31.6	28.3	33.1	21.9	33.4	30.4	25.2	29.5
Donor HbA_{1c} or other measure of blood glucose control	4.4	5.7	5.2	5.8	5	5.6	5.7	5.4
Origin/source of islets[b]	IIDP	Prodo	IIDP	Prodo	IIDP	Prodo	IIDP	IIDP
Islet isolation centre	Imagine Pharma	Prodo	Wisconsin Institution	Prodo	Southern California Islet Cell Resource Center	Prodo	Imagine Pharma	Southern California Islet Cell Resource Center
Donor history of diabetes? Please select yes/no from drop down list	No	No	No	No	No	No	No	No
**If Yes, complete the next two lines if this information is available**								
Diabetes duration (years)								
Glucose-lowering								

therapy at time of death[c]								
**RECOMMENDED INFORMATION**								
Donor cause of death	Stroke	Anoxia	Anoxia	Stroke	Head Trauma	Anoxia	Stroke	Head trauma
Warm ischaemia time (h)								
Cold ischaemia time (h)	11.3	N/A	6	N/A	1.2	N/A	5.2	6.2
Estimated purity (%)	85	90	95	95	80	90	75	80
Estimated viability (%)	85	95	98	95	97	95	90	95
Total culture time (h)[d]								
Glucose-stimulated insulin secretion or other functional measurement[e]								
Handpicked to purity? Please select yes/no from drop down list	No	No	No	No	No	No	No	No
Additional notes								

Islet preparation	25	26	27	28	29	30	31	32
**MANDATORY INFORMATION**								
Unique identifier	HP22362	HP22310	Samn29657580	HP22169	Samn29494177	HP18330	HP22105	Samn27619977

Donor age (years)	51	68	40	46	18	53	58	37
Donor sex (M/F)	M	M	M	M	M	F	F	M
Donor BMI (kg/m^2)	24.9	30.7	23	30	25.3	26.3	28	31.3
Donor HbA_{1c} or other measure of blood glucose control	5.3	5.4	5.2	5.6	5	5.4	5.1	np
Origin/source of islets[b]	Prodo	Prodo	IIDP	Prodo	IIDP	Prodo	Prodo	IIDP
Islet isolation centre	Prodo	Prodo	University of Pennsylvania	Prodo	Southern California Islet Cell Resource Center	Prodo	Prodo	Southern California Islet Cell Resource Center
Donor history of diabetes? Please select yes/no from drop down list	No	No	No	No	No	No	No	No
**If Yes, complete the next two lines if this information is available**								
Diabetes duration (years)								
Glucose-lowering therapy at time of death[c]								
**RECOMMENDED INFORMATION**								
Donor cause of death	Stroke	Anoxia	Head trauma	Head trauma	Head trauma	Anoxia	Head Trauma	Head Trauma
Warm ischaemia time (h)								
Cold ischaemia time (h)	N/A	N/A	11.5	N/A	6	N/A	N/A	6.4
Estimated purity (%)	85	90	90	85	80	90	90	90
Estimated viability (%)	95	95	95	95	98	95	95	96

Total culture time (h)[d]								
Glucose-stimulated insulin secretion or other functional measurement[e]								
Handpicked to purity? Please select yes/no from drop down list	No	No	No	No	No	No	No	No
Additional notes								

**Islet preparation**	**33**	**34**	**35**	**36**	**37**	**38**	**39**	**40**
**MANDATORY INFORMATION**								
Unique identifier	HP22095	HP22089	HP22076	HP22065	HP22068	HP22052	H253	Samn52216388
Donor age (years)	33	48	36	61	20	52	31	38
Donor sex (M/F)	M	M	M	M	M	M	M	F
Donor BMI (kg/m^2)	25.9	19	23.2	22.4	20.8	25.3	33	32.4
Donor HbA_{1c} or other measure of blood glucose control	5.4	5.3	5.3	5.2	4.6	4.2	5.7	5.8
Origin/source of islets[b]	Prodo	Prodo	Prodo	Prodo	Prodo	Prodo	Prodo	U Chicago
Islet isolation centre	Prodo	Prodo	Prodo	Prodo	Prodo	Prodo	Prodo	U Chicago
Donor history of diabetes? Please select yes/no from drop down list	No	No	No	No	No	No	No	No
**If Yes, complete the next two lines if this information is available**								

Diabetes duration (years)								
Glucose-lowering therapy at time of death[c]								
**RECOMMENDED INFORMATION**								
Donor cause of death	Head Trauma	Stroke	Anoxia	Anoxia	Head Trauma	CVA	Anoxi a	Stroke
Warm ischaemia time (h)								
Cold ischaemia time (h)	N/A	N/A	N/A	N/A	N/A	N/A	N/A	5.5
Estimated purity (%)	90	87	90	90	90	90	85	80
Estimated viability (%)	95	95	95	95	95	95	N/A	95
Total culture time (h)[d]								
Glucose-stimulated insulin secretion or other functional measurement[e]								
Handpicked to purity? Please select yes/no from drop down list	No	No	No	No	No	No	No	No
Additional notes								

**Islet preparation**	**41**	**42**	**43**	**44**	**45**	**46**	**47**	**48[a]**
**MANDATORY INFORMATION**								
Unique identifier	R608	R603	R601	HP25249	R595	Samn50550022	Samn50439434	Samn49948188

Donor age (years)	55	41	71	69	57	49	21	54
Donor sex (M/F)	M	M	F	F	M	M	M	M
Donor BMI (kg/m^2)	18.1	29.5	29.5	28	33.1	40.9	26.9	31.6
Donor HbA_{1c} or other measure of blood glucose control	5	5.8	5.2	5.5	5.5	5.3	5.1	5.8
Origin/source of islets[b]	Edmonton	Edmonton	Edmonton	Prodo	Edmonton	IIDP	IIDP	IIDP
Islet isolation centre	Edmonton	Edmonton	Edmonton	Prodo	Edmonton	Imagine Islet Center	University of Pennsylvania Islet Transplant Center	Imagine Islet Center
Donor history of diabetes? Please select yes/no from drop down list	No	No	No	No	No	No	No	No
**If Yes, complete the next two lines if this information is available**								
Diabetes duration (years)								
Glucose-lowering therapy at time of death[c]								
**RECOMMENDED INFORMATION**								
Donor cause of death	N/A	N/A	N/A	Stoke	N/A	Stroke	Anoxia	Stroke
Warm ischaemia time (h)								
Cold ischaemia time (h)	N/A	N/A	N/A	N/A	N/A	9.9	2.9	2.8
Estimated purity (%)	90	85	95	85	90	85	90	80
Estimated viability (%)	N/A	N/A	N/A	95	N/A	92	95	90

Total culture time (h)[d]								
Glucose-stimulated insulin secretion or other functional measurement[e]								
Handpicked to purity? Please select yes/no from drop down list	No	No	No	No	No	No	No	No
Additional notes								

**Islet preparation**	**49**	**50**	**51**	**52**	**53**	**54**	**55**	**56**
**MANDATORY INFORMATION**								
Unique identifier	R589	R592	Samn48756137	R583	HP24265	Hp25113	HP 25108	Samn47319182
Donor age (years)	55	48	35	33	47	45	42	21
Donor sex (M/F)	M	M	F	M	M	M	M	M
Donor BMI (kg/m^2)	24.2	33.2	31.6	23.1	27.8	29.1	31.4	24.5
Donor HbA_{1c} or other measure of blood glucose control	5.4	5.5	5.5	5.7	5.3	5.9	5.3	5.3
Origin/source of islets[b]	Edmonton	Edmonton	IIDP	Edmonton	Prodo	Prodo	Prodo	IIDP
Islet isolation centre	Edmonton	Edmonton	Imagine Islet Center	Edmonton	Prodo	Prodo	Prodo	University of Pennsylvania Islet Transplant Center

Donor history of diabetes? Please select yes/no from drop down list	No	No	No	No	No	No	No	No
**If Yes, complete the next two lines if this information is available**								
Diabetes duration (years)								
Glucose-lowering therapy at time of death[c]								
**RECOMMENDED INFORMATION**								
Donor cause of death	N/A	N/A	Stroke	N/A	Anoxia	Stroke	Strok e	Stroke
Warm ischaemia time (h)								
Cold ischaemia time (h)	N/A	N/A	12.76	N/A	N/A	N/A	N/A	16.8
Estimated purity (%)	95	95	90	95	85	80	90	90
Estimated viability (%)	N/A	N/A	95	N/A	95	95	95	90
Total culture time (h)[d]								
Glucose-stimulated insulin secretion or other functional measureme nt[e]								
Handpicked to purity? Please select yes/no from drop down list	No	No	No	No	No	No	No	No
Additional notes								

Islet preparation	57	58	59	60	61	62	63	64
**MANDATORY INFORMATION**								
Unique identifier	Samn47290391	Hp25038	Hp25030	Samn46400497	HP25007	Samn44655124	Samn44416316	HP24284
Donor age (years)	54	40	52	59	57	23	64	55
Donor sex (M/F)	F	M	F	M	M	F	F	F
Donor BMI (kg/m^2)	30.9	25.5	28.4	27.4	30.4	32.5	23.4	23.3
Donor HbA_{1c} or other measure of blood glucose control	4.7	5.6	5.2	5.3	5.3	5.5	5	5.5
Origin/source of islets[b]	IIDP	Prodo	Prodo	IIDP	Prodo	IIDP	IIDP	Prodo
Islet isolation centre	University of Arizona, Institute for Cellular Transplantation	Prodo	Prodo	VCU Islet Cell Processing Lab Institution	Prodo	Wisconsin Institution	Southern California Islet Cell Resource Center, City of Hope Institution	Prodo
Donor history of diabetes? Please select yes/no from drop down list	No	No	No	No	No	No	No	No
**If Yes, complete the next two lines if this information is available**								
Diabetes duration (years)								
Glucose-lowering therapy at time of death[c]								
**RECOMMENDED INFORMATION**								

Donor cause of death	Stroke	Stroke	Anoxia	Stroke	Head Trauma	Head Trauma	Head Trauma	Stroke
Warm ischaemia time (h)								
Cold ischaemia time (h)	4.4	N/A	N/A	17	N/A	5.15	74	N/A
Estimated purity (%)	95	80	90	95	95	85	85	85
Estimated viability (%)	92	95	95	95	95	98	100	95
Total culture time (h)[d]								
Glucose-stimulated insulin secretion or other functional measurement[e]								
Handpicked to purity? Please select yes/no from drop down list	No	No	No	No	No	No	No	No

**Islet preparation**	**65**	**66**	**67**	**68**	**69**	**70**	**71**	**72**
**MANDATORY INFORMATION**								
Unique identifier	Samn44073850	Samn43823987	HP24269	HP24254	Samn43474066	HP24238	Samn43358320	R549
Donor age (years)	39	49	43	57	42	69	41	54
Donor sex (M/F)	M	F	M	M	M	F	M	M
Donor BMI (kg/m^2)	27.1	37.6	27.1	28.7	24.4	28	28.7	26.5
Donor HbA_{1c} or other measure of blood glucose control	5.3	5.6	5.5	5.9	4.6	5.5	5.3	5.1

Origin/source of islets[b]	IIDP	IIDP	Prodo	Prodo	IIDP	Prodo	IIDP	Edmonton
Islet isolation centre	University of Arizona, Institute for Cellular Transplantation	Southern California Islet Cell Resource Center, City of Hope Institution	Prodo	Prodo	Southern California Islet Cell Resource Center, City of Hope Institution	Prodo	Columbia University Islet Core	Edmonton
Donor history of diabetes? Please select yes/no from drop down list	No	No	No	No	No	No	No	No
**If Yes, complete the next two lines if this information is available**								
Diabetes duration (years)								
Glucose-lowering therapy at time of death[c]								
**RECOMMENDED INFORMATION**								
Donor cause of death	Head Trauma	Stroke	Head Trauma	Stroke	Stroke	Stroke	Head Trauma	N/A
Warm ischaemia time (h)								
Cold ischaemia time (h)	7.9	8.6	N/A	N/A	1.8	N/A	9.55	N/A
Estimated purity (%)	90	90	90	90	90	85	95	75
Estimated viability (%)	95	95	95	95	95	95	95	N/A
Total culture time (h)[d]								
Glucose-stimulated insulin secretion or other functional measurement[e]								
Handpicked to purity? Please select yes/no from drop down list	No	No	No	No	No	No	No	No

Islet preparation	73	74	75	76	77	78	79	80
**MANDATORY INFORMATION**								
Unique identifier	Samn42877906	HP24194	Samn41611427	Samn41393792	HP24133	HP25161	Samn41247465	Samn41142257
Donor age (years)	42	52	28	27	58	57	43	39
Donor sex (M/F)	M	F	F	M	M	F	M	F
Donor BMI (kg/m^2)	24.4	22.8	35	30.1	32	28.4	32.5	36.6
Donor HbA_{1c} or other measure of blood glucose control	4.6	5.4	5.8	5.5	5.6	5.6	4.9	6
Origin/source of islets[b]	IIDP	Prodo	IIDP	IIDP	Prodo	Prodo	IIDP	IIDP
Islet isolation centre	Imagine Islet Center, Imagine Pharma	Prodo	Nationwide Children's Islet Biology Center Institution	Nationwide Children's Islet Biology Center Institution	Prodo	Prodo	Columbia University Islet Core Institution	Southern California Islet Cell Resource Center, City of Hope Institution
Donor history of diabetes? Please select yes/no from drop down list	No	No	No	No	No	No	No	No
**If Yes, complete the next two lines if this information is available**								
Diabetes duration (years)								
Glucose-lowering therapy at time of death[c]								
**RECOMMENDED INFORMATION**								

Donor cause of death	Stroke	Stroke	Anoxia	Anoxia	Stroke	Anoxia	Anoxia	Storke
Warm ischaemia time (h)								
Cold ischaemia time (h)	1.8	N/A	6	8.5	N/A	N/A	8.5	11.1
Estimated purity (%)	90	90	80	90	90	90	90	90
Estimated viability (%)	N/A	95	85	85	95	95	80	95
Total culture time (h)[d]								
Glucose-stimulated insulin secretion or other functional measurement[e]								
Handpicked to purity? Please select yes/no from drop down list	No	No	No	No	No	No	No	No

**Islet preparation**	**81**	**82**	**83**	**84**	**85**	**86**		
**MANDATORY INFORMATION**								
Unique identifier	Samn41071408	Samn41042507	Samn40963318	HP24095	HP24086	SAMN58977884		
Donor age (years)	43	37	48	44	32	45		
Donor sex (M/F)	M	M	M	M	M	M		
Donor BMI (kg/m^2)	38.4	31.3	36.5	28.6	23.6	25.9		
Donor HbA_{1c} or other measure of blood	5.5	5.6	5.6	5.7	5.7	5.8		

glucose control								
Origin/source of islets[b]	IIDP	IIDP	IIDP	Prodo	Prodo	IIDP		
Islet isolation centre	Nationwide Children's Islet Biology Center	University of Pennsylvania Islet Transplant Center Institution	University of Wisconsin Human Islet Core Institution	Prodo	Prodo	UPENN		
Donor history of diabetes? Please select yes/no from drop down list	No	No	No	No	No	No		
**If Yes, complete the next two lines if this information is available**								
Diabetes duration (years)								
Glucose-lowering therapy at time of death[c]								
**RECOMMENDED INFORMATION**								
Donor cause of death	Anoxia	Anoxia	Anoxia	Stroke	Anoxia	Anoxia		
Warm ischaemia time (h)								
Cold ischaemia time (h)	6	9.4	9	N/A	N/A	2		
Estimated purity (%)	85	90	95	90	85	90		
Estimated viability (%)	85	96	98	95	95	95		
Total culture time (h)[d]								
Glucose-stimulated insulin secretion or other functional measurement[e]								
Handpicked to purity? Please select yes/no from	No	No	No	No	No	No		

drop down list								

**Islet preparation**	**87**	**88**	**89**	**90**	**91**	**6**	**7**	**8[a]**
**MANDATORY INFORMATION**								
Unique identifier	HP26120	Samn57528403	HP26111	HP26124	HP26126			
Donor age (years)	38	44	64	36	43			
Donor sex (M/F)	M	F	F	M	M			
Donor BMI (kg/m^2)	22.2	32.4	26.4	28.7	17.2			
Donor HbA_{1c} or other measure of blood glucose control	5.9	5.1	4.7	5.6	5.2			
Origin/source of islets[b]	Prodo	IIDP	Prodo	Prodo	Prodo			
Islet isolation centre	Prodo	University of Arizon	Prodo	Prodo	Prodo			
Donor history of diabetes? Please select yes/no from drop down list	No	No	No	No	No			
**If Yes, complete the next two lines if this information is available**								
Diabetes duration (years)								
Glucose-lowering therapy at time of death[c]								
**RECOMMENDED INFORMATION**								
Donor cause of death	Head Trauma	Stroke	Head Trauma	Head trauma	Anoxic			
Warm ischaemia time (h)								
Cold ischaemia time (h)		11.4	N/A	N/A	N/A			
Estimated purity (%)	90	95	95	90	90			

Estimated viability (%)	95	95	95	95	95			
Total culture time (h)[d]								
Glucose-stimulated insulin secretion or other functional measurement[e]								
Handpicked to purity? Please select yes/no from drop down list	No	No	No	No	No			
